# Highly efficient ethylene production via electrocatalytic hydrogenation of acetylene under mild conditions

**DOI:** 10.1038/s41467-021-27372-8

**Published:** 2021-12-06

**Authors:** Suheng Wang, Kelechi Uwakwe, Liang Yu, Jinyu Ye, Yuezhou Zhu, Jingting Hu, Ruixue Chen, Zheng Zhang, Zhiyou Zhou, Jianfeng Li, Zhaoxiong Xie, Dehui Deng

**Affiliations:** 1 State Key Laboratory for Physical Chemistry of Solid Surfaces, Collaborative Innovation Center of Chemistry for Energy Materials, College of Chemistry and Chemical Engineering, Xiamen University, Xiamen, 361005 China; 2 State Key Laboratory of Catalysis, Collaborative Innovation Center of Chemistry for Energy Materials, Dalian Institute of Chemical Physics, Chinese Academy of Sciences, Dalian, 116023 China; 3 University of Chinese Academy of Sciences, Beijing, 100049 China

**Keywords:** Electrocatalysis, Catalytic mechanisms, Materials for energy and catalysis

## Abstract

Renewable energy-based electrocatalytic hydrogenation of acetylene to ethylene (E-HAE) under mild conditions is an attractive substitution to the conventional energy-intensive industrial process, but is challenging due to its low Faradaic efficiency caused by competitive hydrogen evolution reaction. Herein, we report a highly efficient and selective E-HAE process at room temperature and ambient pressure over the Cu catalyst. A high Faradaic efficiency of 83.2% for ethylene with a current density of 29 mA cm^−2^ is reached at −0.6 V vs. the reversible hydrogen electrode. In-situ spectroscopic characterizations combined with first-principles calculations reveal that electron transfer from the Cu surface to adsorbed acetylene induces preferential adsorption and hydrogenation of the acetylene over hydrogen formation, thus enabling a highly selective E-HAE process through the electron-coupled proton transfer mechanism. This work presents a feasible route for high-efficiency ethylene production from E-HAE.

## Abstract (web summary)

This work achieves highly active and selective electrocatalytic hydrogenation of acetylene to ethylene (HAE) under mild conditions over Cu catalyst to replace the classic thermocatalytic HAE route requiring harsh conditions and costly H_2_.

## Introduction

Integrated with technology of acetylene production from natural gas or coal, selective hydrogenation of acetylene to ethylene (HAE) has emerged as a promising non-oil route for producing ethylene, which is one of the most important building blocks in chemical synthesis^1–3^. Thermocatalytic HAE typically requires high temperatures above 200 °C and high pressures of around 5 bar^4–8^, and thus is highly energy demanding. Besides, large amount of H_2_ consumption makes the process even more costly. Moreover, the selectivity of ethylene is also hardly controllable owing to the undesired but facile excessive hydrogenation of acetylene to ethane under the harsh reaction conditions in the thermocatalytic HAE^9–12^. Therefore, it is of great significance to develop a more economical, energy-efficient, and highly selective route for the HAE process.

**Fig. 1:**
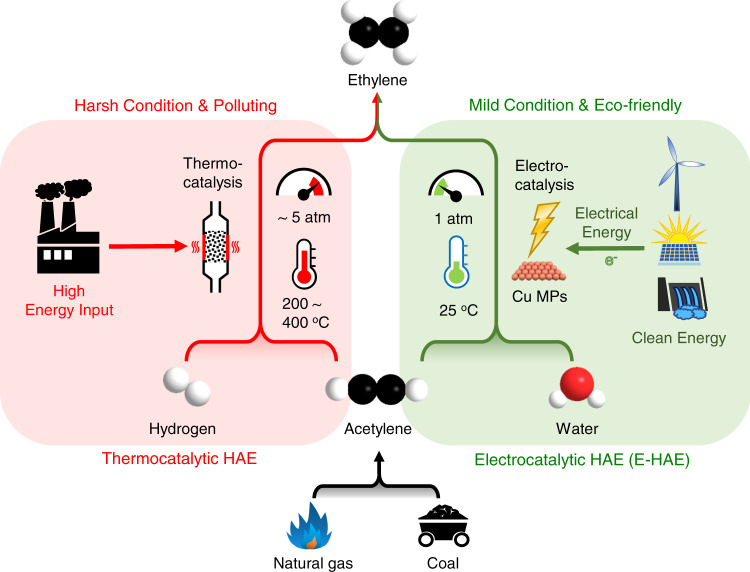
The features of the electrocatalytic HAE (E-HAE) process compared with the traditional thermocatalytic HAE process. The E-HAE process is more economical and environmental-friendly, operatable under mild reaction conditions with inexpensive water as hydrogen source, in contrast to the thermocatalytic process, which requires harsh reaction conditions and an extra supply of H_2_.

Herein, we report a highly efficient and selective electrocatalytic HAE (E-HAE) process at room temperature and ambient pressure over carbon-supported Cu microparticles (MPs). In contrast to the thermocatalytic path, this process is advantageous in being operatable under mild conditions and is environmental-friendly in combination with electroreduction of water based on renewable electricity, in which hydrogen is in-situ generated for the reduction reaction so that extra supply of H_2_ can be avoided (Fig. 1), which emerges as an attractive route for semihydrogenation of alkynes^13–15^. By optimizing the Cu catalyst to expose more active facets, preferential adsorption and hydrogenation of acetylene is facilitated against hydrogen adsorption and evolution. In combination with adjusting the electrode potential to modulate the selectivity of the reduction products, excessive hydrogenation of acetylene to ethane is fully avoided at cathode potentials above −0.6 V vs. reversible hydrogen electrode (RHE). By coating the carbon support with a microporous gas diffusion layer (GDL) to promote mass transfer, a high Faradaic efficiency of 83.2% for ethylene production (${{{{{{\rm{FE}}}}}}}_{{{{{{{\rm{C}}}}}}}_{2}{{{{{{\rm{H}}}}}}}_{4}}$) is achieved with an overall current density (*j*) of 29 mA cm^−2^ at −0.6 V vs. RHE. The geometric current density for ethylene formation (${j}_{{{{{{{\rm{C}}}}}}}_{2}{{{{{{\rm{H}}}}}}}_{4}}$) reaches 26.7 mA cm^−2^ at −0.7 V vs. RHE. By using continuous gas-flow method in the E-HAE process, the faradaic efficiency and the overall formation rate of ethylene are largely improved compared with those in closed reaction systems of previous reports^16–21^. In situ spectroscopic characterizations combined with density functional theory (DFT) calculations demonstrate that electron transfer from the Cu surface to adsorbed acetylene promotes the adsorption and hydrogenation of the acetylene, while suppressing the competitive hydrogen evolution reaction (HER) and facilitating ethylene desorption, thereby resulting in highly selective ethylene production. The electrochemical hydrogenation proceeds via the electron-coupled proton transfer pathways, which require higher activation energies for further hydrogenation steps than the desorption of the generated ethylene, so that the excessive hydrogenation to ethane is effectively inhibited. This process provides a green route for industrial production of C_2_H_4_ from C_2_H_2_ beyond the removal of C_2_H_2_ impurities in C_2_H_4_^14,15^.

## Results and discussion

### Structure and E-HAE performance of the Cu catalyst

**Fig. 2:**
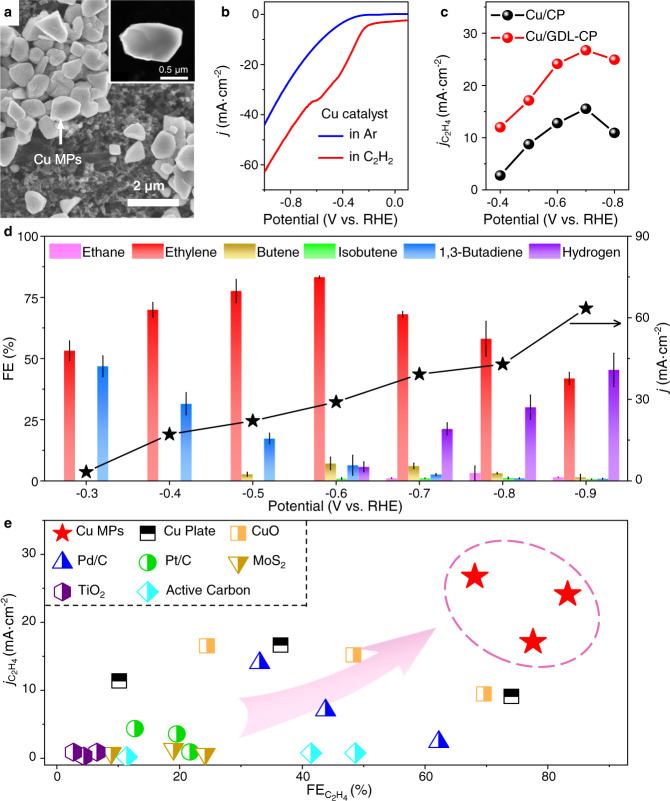
Characterizations of the structure and E-HAE performance of the Cu catalyst. **a** SEM and HAADF-STEM (inset) image of the catalyst. **b** Linear sweep voltammetric curves in Ar-saturated and C_2_H_2_-saturated solution. The scanning rate is 10 mV s^−1^. **c** Comparison in the geometric current density of ethylene (${j}_{{{{{{{\rm{C}}}}}}}_{2}{{{{{{\rm{H}}}}}}}_{4}}$) over the Cu microparticles (MPs) loaded on pristine carbon papers (Cu/CP) and carbon papers coated with the microporous gas diffusion layer (Cu/GDL-CP). **d** FEs of the E-HAE products and the current densities at different potentials. Error bars correspond to the standard deviation. **e** Comparison in the FE and the geometric current density for ethylene production from E-HAE over different catalysts, which were tested in this work. All experiments were conducted in 1 M KOH solution at 25 °C. Source data are provided as a Source Data file.

The Cu MPs were prepared via a facile solvothermal route in ethylene glycol (EG) using CuSO_4_·5H_2_O as precursors. The morphology of the Cu catalyst was characterized by scanning electron microscopy (SEM, Fig. 2a), high angle annular dark-field scanning transmission electron microscopy (HAADF-STEM) (Fig. 2a) and transmission electron microscopy (TEM) (Supplementary Fig. 1), which show that the Cu MPs are uniformly distributed with diameters of around 1 μm. Powder X-ray diffraction (XRD) characterizations show that the Cu MPs with face-centered cubic (fcc) structure mainly expose (111), (100), and (110) facets, but possessing more (100) and (110) facets than the commercial Cu bulk according to their normalized peak intensities (Supplementary Fig. 2).

Electrochemical tests were carried out to measure the E-HAE performance of the Cu MPs loaded on GDL-coated carbon paper (Cu/GDL-CP). The linear sweep voltammetry (LSV) was performed to assess the E-HAE activity of the catalyst. In Ar-saturated solution, the current density is close to zero even at an electrode potential of −0.3 V vs. RHE with or without *iR* compensation, indicating a poor HER activity of the catalyst. As the solution is saturated by C_2_H_2_, the current density increases remarkably with an onset potential of around −0.24 and −0.15 V before and after the *iR* compensation, respectively (Fig. 2b and Supplementary Fig. 3), owing to the contribution of electrocatalytic hydrogenation of acetylene. The different activity of the Cu MPs for the HER and E-HAE is further confirmed by remeasuring the LSV curves using rotating disk electrode (RDE) instead with loaded catalyst, which present a similar difference in the onset potentials of the two processes (Supplementary Fig. 3), thus demonstrating the higher activity of the catalyst for the E-HAE process.

The microporous GDL, consisting of polytetrafluoroethylene microspheres and hydrophobic carbon particles coated on carbon paper (Supplementary Figs. 4 and 5), plays a significant role in improving the E-HAE reaction efficiency by promoting mass transfer. The GDL is favorable for the formation of gas–liquid–solid interfaces by increasing the hydrophobicity and surface area of electrodes. As indicated in the contact angle tests (Supplementary Fig. 6), the electrode with Cu/GDL-CP is more hydrophobic than the electrode with Cu MPs loaded on pristine carbon paper (Cu/CP) (Supplementary Fig. 7). The BET specific surface area (SSA) measurements show that the GDL also remarkably increases the SSA of the CP from 0.37 to 4.55 m^2^ g^−1^, which is favorable for constructing gas–liquid–solid interfaces. Though the Cu/GDL-CP and Cu/CP have similar electrochemical active surface areas (ECSAs), which were measured as 6.2 and 6.4 cm^2^, respectively (Supplementary Fig. 8), the geometric current densities of acetylene consumption (${j}_{{{{{{{\rm{C}}}}}}}_{2}{{{{{{\rm{H}}}}}}}_{2}}$) and ethylene formation (${j}_{{{{{{{\rm{C}}}}}}}_{2}{{{{{{\rm{H}}}}}}}_{4}}$) on the Cu/GDL-CP are always notably higher than those on the Cu/CP (Supplementary Fig. 9, Fig. 2c). Moreover, the FEs of HER on the Cu/GDL-CP at different potentials are obviously lower than those on the Cu/CP, especially at potentials below −0.6 V vs. RHE (Supplementary Fig. 10). These results indicate that the GDL can effectively promote the E-HAE process and suppress the HER.

The distribution of products strongly depends on the electrode potential (Fig. 2d). At −0.3 V vs. RHE, ethylene and 1,3-butadiene are the main products with FEs of around 53% and 47%, respectively. With the decrease in potential, the ${{{{{{\rm{FE}}}}}}}_{{{{{{{\rm{C}}}}}}}_{2}{{{{{{\rm{H}}}}}}}_{4}}$ grows gradually and reaches a maximum of 83.2% at −0.6 V with an overall current density of 29 mA cm^−2^, while the FE of 1,3-butadiene decreases significantly to below 10% with few butene generated. Note that the excessive hydrogenation of acetylene to ethane is fully avoided at cathode potentials above −0.6 V. As the potential becomes more negative, H_2_ and some deep hydrogenated products such as ethane begin to form, leading to decreased ${{{{{{\rm{FE}}}}}}}_{{{{{{{\rm{C}}}}}}}_{2}{{{{{{\rm{H}}}}}}}_{4}}$. These results show that the electrochemical reductivity strongly depends on the electrode potential, which determines the selectivity of the reduction products. At the potential of −0.7 V, the rate of acetylene consumption (*r*_ace_) and geometric ${j}_{{{{{{{\rm{C}}}}}}}_{2}{{{{{{\rm{H}}}}}}}_{4}}$ reach maxima of 0.59 mmol cm^−2^ h^−1^ and 26.7 mA cm^−2^, respectively (Supplementary Fig. 11 and Fig. 2c), with acetylene conversion of around 4.9%. Compared with other tested and reported catalysts, the Cu MPs in this work exhibits the highest ${{{{{{\rm{FE}}}}}}}_{{{{{{{\rm{C}}}}}}}_{2}{{{{{{\rm{H}}}}}}}_{4}}$ and geometric ${j}_{{{{{{{\rm{C}}}}}}}_{2}{{{{{{\rm{H}}}}}}}_{4}}$ for the E-HAE (Fig. 2e, Supplementary Table 1)^16–18^. Moreover, by tandemly connecting more electrolytic cells with adjusted flow rate to 2.4 mL/min, the conversion of acetylene can be further increased to 9.3% in a single-cell system, and to 17.6% in a double-cell system and 23.3% in a triple-cell system (Supplementary Fig. 12), where the reaction rate of acetylene can still reach 0.45 mmol cm^−2^ h^−1^ with ethylene selectivity of over 80% and ethylene Faradaic efficiency of around 50%. This exhibits the potential of the E-HAE process for industrial applications.

Long-term stability of the Cu catalyst was measured at the potential of −0.6 V vs. RHE. The ${{{{{{\rm{FE}}}}}}}_{{{{{{{\rm{C}}}}}}}_{2}{{{{{{\rm{H}}}}}}}_{4}}$ can be well-maintained above 70% for over 100 h with a current density of around 12 mA cm^−2^ (Supplementary Fig. 13). The activity loss may be due to partial blocking of active sites by the formed polyacetylene layer on the catalyst surface, as detected by TEM and Raman spectroscopy showing the feature peaks of polyacetylene at 1102 and 1492 cm^−1^ (Supplementary Figs. 14 and 15)^22^.

### Electronic property of the Cu catalyst

**Fig. 3:**
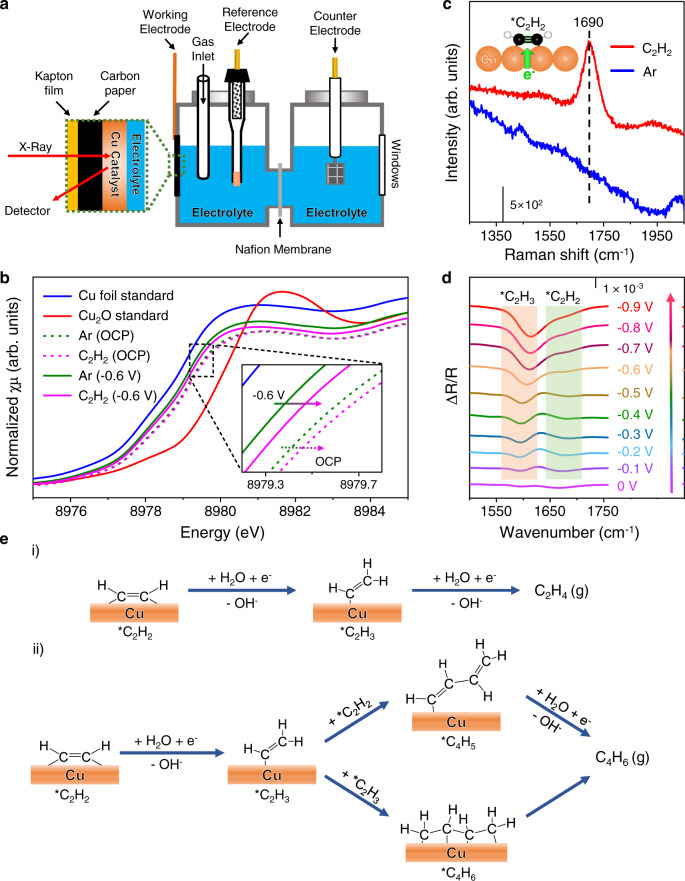
In-situ spectroscopic characterizations of the Cu catalyst and the reaction intermediates. **a** The schematic illustration of in-situ XANES electrolytic cell. **b** The in-situ Cu K-edge XANES of the catalyst in Ar-saturated and C_2_H_2_-saturated solution at OCP and −0.6 V, compared with those of Cu foil and Cu_2_O standard sample. **c** The in-situ Raman spectra of the catalyst in Ar-saturated solution and C_2_H_2_-saturated solution. **d** The in-situ ATR-FTIR spectra during the E-HAE process at potentials from 0 to −0.9 V in C_2_H_2_-saturated solution. The reflection intensity is decreased due to infrared absorption of surface species; thus, the generation of species leads to a negative peak. **e** The proposed reaction routes for the formation of (i) ethylene and (ii) 1,3-butadiene. All the potentials in the figures are referenced to the RHE. The in-situ XANES and Raman experiments were conducted in 1 M KOH solution at 25 °C. The in-situ ATR-FTIR experiments were conducted in 0.1 M KOH solution at 25 °C. Source data are provided as a Source Data file.

In-situ X-ray absorption fine structure (XAFS) experiments were performed to investigate the electronic and geometric properties of the Cu catalyst^23^. The reaction was carried out in a specially designed H-type cell (Fig. 3a and Supplementary Fig. 16). The profile of the X-ray absorption near-edge structure (XANES) spectra of the Cu K-edge under different conditions all resemble that of the Cu foil with white lines slightly shifted to higher energies, thus indicating a mild oxidization state of the Cu catalyst which may be attributed to exposure of the catalyst to air (Fig. 3b). At cathodic potential of −0.6 V vs. RHE in Ar-saturated electrolyte, the white line shifts back to a lower energy in contrast to that at open-circuit potential (OCP), which indicates a reduction of the catalyst surface by the electrode potential. In C_2_H_2_-saturated electrolyte, the white lines shift again to a higher energy relative to those in the Ar-saturated electrolyte at −0.6 V or OCP (Fig. 3b), which was also observed and confirmed at other reaction potentials of −0.4 and −0.8 V (Supplementary Fig. 17).The DFT-simulated Cu K-edge XANES spectra also show that all the absorption edges of the surface Cu atoms on Cu(100), (110), and (111) shift to higher binding energies after the adsorption of C_2_H_2_ on the surfaces (Supplementary Fig. 18). This indicates that the C_2_H_2_ adsorption on the surface leads to increased oxidation state of the catalyst owing to the electron transfer from Cu to antibonding π orbital of the adsorbed C_2_H_2_ (*C_2_H_2_)^24^. This result is also confirmed by in-situ Raman spectroscopy characterizations at OCP (Fig. 3c), in which the significant peak at 1690 cm^−1^ assigned to the carbon–carbon triple bond of *C_2_H_2_ on the Cu surface in C_2_H_2_-saturated solution, is remarkably red-shifted relative to that of the gas phase C_2_H_2_^25^. Thereby, the C–C bond of the *C_2_H_2_ is likely weakened and activated due to the filling of antibonding orbital by the electrons transferred from Cu. The corresponding Fourier-transformed extended XAFS spectra in the R-space for the Cu MPs catalyst before and after the reaction, are almost identical and are quite similar with that for the Cu foil (Supplementary Fig. 19), thus indicating a high stability of the catalyst during the E-HAE process.

### Reaction intermediates in the E-HAE

To investigate the reaction intermediates during the E-HAE, we carried out the in-situ attenuated total reflectance-Fourier transform infrared (ATR-FTIR) spectroscopy characterizations, which is highly sensitive to surface species^26,27^. The in-situ ATR-FTIR spectrum at different potentials are plotted in Fig. 3d. At −0.1 V vs. RHE, a positive peak at 1630 cm^−1^ and two negative peaks at 1594 and 1670 cm^−1^ were observed. The positive peak at 1630 cm^−1^ is assigned to the desorbed H_2_O from the catalyst surface^28^. The negative band at 1594 cm^−1^ is assigned to the generated *CH = CH_2_ (*C_2_H_3_), a reaction intermediate of C_2_H_2_ reduction^29,30^. The peak at 1670 cm^−1^ is assigned to the *C_2_H_2_ on the Cu surface, which is close to that of the in-situ Raman spectrum (Fig. 3c). As the electrode potential becomes more negative, the peak of *C_2_H_3_ at 1594 cm^−1^ increases prominently, indicating that hydrogenation of the *C_2_H_2_ to *C_2_H_3_ is strongly related to the electrode potential and the *C_2_H_3_ is the key intermediate for the formation of C_2_H_4_. The evolution of surface species was further validated using a reversed sweeping of potential from −0.9 to 0.1 V with an interval of 0.2 V (Supplementary Fig. 20), which shows a reversed change in the peak intensities. According to these results, the possible reaction routes in the E-HAE on the Cu catalyst are proposed (Fig. 3e): acetylene is firstly hydrogenated to *C_2_H_3_ intermediate, which then undergoes further hydrogenation to ethylene, or coupling and hydrogenation to 1,3-butadiene by-product.

### Understanding the reaction mechanism

**Fig. 4:**
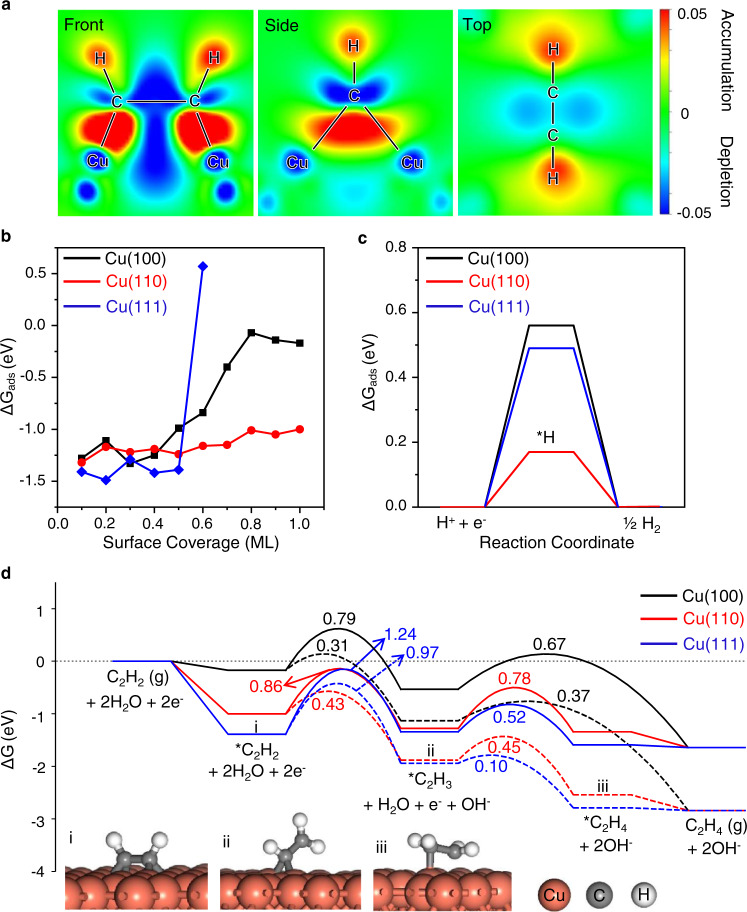
Theoretical insight into the reaction mechanism. **a** Charge density difference of the adsorbed C_2_H_2_ on the Cu surface at front, side and top views. Charge accumulation and depletion regions are shown in red and blue respectively. **b** Adsorption free energy of C_2_H_2_ on Cu(100), (110), and (111) surfaces at different coverages at 25 °C. **c** The free energy profile diagram of hydrogen evolution reaction at 0 V vs. RHE, 25 °C on the covered Cu surfaces. **d** Free energy diagram for the formation of C_2_H_4_ on Cu(100), Cu(110), and Cu(111) covered surfaces at 0 and −0.6 V vs. RHE, 25 °C. The solid lines represent the potential energy surfaces at 0 V, while the dashed lines represent the potential energy surfaces at −0.6 V. For a clearer description of the reaction states only the species concerned are shown in the inserted screenshots, the neighboring adsorbed species are not shown for simplicity. Source data are provided as a Source Data file.

DFT calculations were carried out to gain further insights into the active sites and the reaction mechanism of the E-HAE process over the Cu catalyst. Three crystal facets of Cu including (111), (100), and (110) were considered according to the XRD analysis (Supplementary Fig. 2). On all these surfaces, C_2_H_2_ prefers to be strongly adsorbed on the hollow sites with much higher adsorption free energies compared with hydrogen adsorption (Supplementary Fig. 21). Bader charge analysis shows a significant decrease in the valence electrons of the Cu atoms with an increase of up to 0.65 electrons on the *C_2_H_2_, thus demonstrating the electron transfer from the Cu surface to the *C_2_H_2_ which is in consistency with the experimental results as discussed above. The electron transfer can be more clearly visualized in the differential charge density of the adsorption structure (Fig. 4a), showing charge depletion areas around the Cu adsorption sites and charge accumulation areas around the *C_2_H_2_ on the Cu surfaces. Considering the strong adsorption of C_2_H_2_, the surface coverage effect of C_2_H_2_ was included in studying the reaction mechanism. Equilibrium coverages of 1.0, 1.0, and 0.5 monolayer C_2_H_2_ was obtained on the Cu(100), (110), and (111) surfaces, respectively, by calculating the adsorption free energy (Δ*G*_ads_) of C_2_H_2_ on the gradually covered surfaces until no further adsorption is favorable (Fig. 4b, Supplementary Fig. 22). The electrochemical hydrogenation reaction mechanism was then investigated on the C_2_H_2_-covered surfaces.

The formation of surface H species (*H) on the C_2_H_2_-covered surfaces is energetically unfavorable with Δ*G*_ads_ of 0.56, 0.17, and 0.49 eV on the Cu(100), (110), and (111) surfaces, respectively (Fig. 4c). In addition, the formation process of *H via electron-coupled proton transfer from the water layer to the C_2_H_2_-covered surfaces also requires high barriers of approximately 0.81, 1.84, and 0.97 eV on the Cu(100), (110), and (111) surfaces, respectively. These results demonstrate that *H can hardly be formed on the C_2_H_2_-covered Cu surfaces, which could explain the poor HER activity at potentials above −0.6 V vs. RHE. This also indicates that the *C_2_H_2_ is not likely hydrogenated by surface *H, but rather via electron-coupled proton transfer from the water layer (Eq. 1), as also reflected by the pH effect on the overall Faradaic efficiencies for acetylene hydrogenation and HER, where a lower pH is more favorable for the hydrogenation of acetylene than the HER (Supplementary Fig. 23).

1 $$\ast {{{{{\rm{C}}}}}}_{{{{{\rm{x}}}}}}{{{{{\rm{H}}}}}}_{{{{{\rm{y}}}}}}+{{{{{\rm{H}}}}}}_{2}{{{{{\rm{O}}}}}}+{{{{{\rm{e}}}}}}^{-}\to \ast {{{{{\rm{C}}}}}}_{{{{{\rm{x}}}}}}{{{{{\rm{H}}}}}}_{{{{{\rm{y+1}}}}}}+{{{{{\rm{OH}}}}}}^{-}$$

The free energy reaction pathways for the formation of C_2_H_4_ on the three Cu surfaces were calculated as shown in Fig. 4d and Supplementary Table 2. Hydrogenation of the *C_2_H_2_ to *C_2_H_3_ is the rate-limiting step on all the three surfaces with barriers of 0.79, 0.86, and 1.24 eV on the (100), (110), and (111) surfaces at 0 V vs. RHE, respectively. The highest barrier for the first hydrogenation step on the Cu(111) surface may be due to the much stronger adsorption of C_2_H_2_ on the surface (Fig. 4b). The second hydrogenation steps all proceed with relatively lower barriers and the generated C_2_H_4_ can be easily desorbed from the surfaces, which are kinetically more favorable than the further hydrogenation step via the electron-coupled proton transfer and thereby suppresses the excessive hydrogenation pathway. Formation of C_4_H_6_ was also investigated which involves both electrochemical hydrogenation steps and non-electrochemical carbon–carbon (C–C) coupling steps, and thus can occur in two pathways: coupling of *C_2_H_3_ and *C_2_H_2_ (First pathway) or two *C_2_H_3_ (Second pathway) (Supplementary Figs. 24 and 25, Supplementary Tables 3 and 4). Both reaction pathways have relatively lower barriers on the Cu(100) surface, which should be due to the relatively weaker adsorption of reaction intermediates on the C_2_H_2_-covered (100) surface.

The high selectivity of C_2_H_4_ against C_4_H_6_ is largely affected by the applied negative potential as shown in Fig. 2d, in contrast with the selectivity at 0 V vs. RHE. This is because the electrochemical hydrogenation steps involve significant electron transfer and are potential-dependent. The applied negative potentials lead to lowered work function of the surface, which could stabilize the transition states (TSs) and thereby reduce the energy barriers^31^. At −0.6 V vs. RHE, the activation energies of the electrochemical hydrogenation steps are significantly reduced compared with those at 0 V, especially for the rate-limiting *C_2_H_2_ hydrogenation to *C_2_H_3_, the activation energies of which on the (100) and (110) surfaces are more reduced and become even comparable with those of other steps (Fig. 4d, Supplementary Figs. 24 and 25, Supplementary Tables 2–4). Therefore, by adjusting the electrode potential, the potential-dependent electrochemical hydrogenation steps can be promoted over the potential-independent non-electrochemical C–C coupling steps, thereby increasing the selectivity of C_2_H_4_ on the Cu catalyst.

In conclusion, we reported a highly efficient electrocatalytic process for acetylene hydrogenation via using the Cu catalyst at room temperature and ambient pressure. By adjusting the electrode potential as well as using the GDL to promote mass transfer, a highest ${{{{{{\rm{FE}}}}}}}_{{{{{{{\rm{C}}}}}}}_{2}{{{{{{\rm{H}}}}}}}_{4}}$ of 83.2% was reached at −0.6 V vs. RHE, and a highest geometric ${j}_{{{{{{{\rm{C}}}}}}}_{2}{{{{{{\rm{H}}}}}}}_{4}}$ of 26.7 mA cm^−2^ was reached at −0.7 V, which are better than those of other tested and previously reported catalysts. Moreover, during the 100-h stability test, the Cu catalyst shows a good performance. The in-situ characterizations and DFT calculations confirm that electron transfer from the Cu surface to the acetylene enhances the adsorption of acetylene against hydrogen, which suppresses the HER while promoting the E-HAE through the electron-coupled proton transfer mechanism. The E-HAE process reported in this work provides new prospect for developing energy-efficient and eco-friendly ways of ethylene production.

## Methods

### Preparation of catalysts

All chemicals were used without further purification. Ethylene glycol (EG) was purchased from Sigma-Aldrich. Copper sulfate pentahydrate (CuSO_4_·5H_2_O), ascorbic acid (AA), NaOH, NaCl, ethanol, and polyvinyl pyrrolidone (PVP) K-30 were bought from Sinopharm Chemical Reagent Co. Ltd. Pt/C was produced by Johnson Matthey. Pd/C was bought from J&K Scientific Ltd. The TiO_2_(P25) was purchased from Degussa. Active carbon was purchased from Sigma-Aldrich. The Cu plate was purchased from Shichun Co. The characterizations of catalysts were presented in Supplementary Figs. 26–29.

To synthesize the Cu MPs, 125 mg CuSO_4_·5H_2_O and 200 mg PVP were dissolved in a flask containing 20 mL EG. Then 0.2 mL 1 M NaCl EG solution was added into the flask. Then the mixture was preheated to 100 °C. Next, another mixture of 1 mL 1 M NaOH and 2.5 mL 0.5 M AA EG solution was injected into the flask for 10 min. Then the mixture was heated for 40 min in 100 °C. Then the Cu MPs were cleaned using a mixture of alcohol and water by centrifuge. Finally, the Cu MPs were kept in alcohol in the freezer of a refrigerator.

The CuO was synthesized by heating the Cu MPs at 500 °C for 1 h under 20% O_2_/Ar (Supplementary Fig. 30). The MoS_2_ catalyst was synthesized by using our previously reported method (Supplementary Fig. 31). Typically, 900 mg (NH_4_)_6_Mo_7_O_24_·4H_2_O was dissolved in 20 mL deionized water. Then, this solution and 10 ml CS_2_ were sealed in a 40 mL stainless-steel autoclave under Ar protection and maintained at 400 °C for 4 h. The Cu plate was cut into “L” shape with a 1 × 1 cm^2^ square and a 0.5 × 1 cm^2^ bar. The Cu plate was polished by silicon carbide papers, followed by alumina polishing powder (1 μm, 0.05 μm).

### Characterization

SEM characterizations were conducted on Hitachi S4800. TEM measurements were conducted on the FEI Tecnai F20 and FEI talos F200X microscopes operated at an accelerating voltage of 200 kV. To prepare the TEM sample, 100 μL Cu MPs ink was dispersed in 3 mL ethanol and treated with ultrasound for 0.5 h; then the solution was dropped on a Cu net for TEM and then dried by a heating light for 10 min. The XRD measurements were carried out on a Rigaku Ultima IV diffractometer with Cu Ka radiation at 40 kV and 30 mA.

### E-HAE reaction performance test

The 4 mg catalysts mixed with 9 μL Nafion solution (5 wt%, Du Pont) and drip-coated on carbon paper with the GDL-CP (AvCarb GDS3250 with micro-porous layer) were used as the working electrode. The carbon paper was cut into an “L” shape with a 1 × 1 cm^2^ square and a 0.5 × 1 cm^2^ bar (Supplementary Fig. 32a, c). Totally, 4 mg Cu MPs were dispersed in 0.5 mL ethanol and were dripped on the 1 × 1 cm^2^ square part, which was dried under a heating light (Supplementary Fig. 32b, d). The electrode is fixed by the electrode holder via the bar. Only the 1 × 1 cm^2^ square part of carbon paper with catalysts was immersed in the electrolyte. New working electrodes were prepared for every electrochemical measurement. The electrode was kept in a vacuum drier before use. Alkaline aqueous solutions with KOH concentration of 0.1, 1, and 3 M were used as the electrolyte. The electrocatalytic performances were measured on a CHI 660e with a H-type electrochemical cell equipped with a flow-controlling device. The anode and cathode electrode chambers were divided by the Nafion-115 membrane. The reference electrode is the Hg/HgO electrode filled with 1 M KOH. All potentials were measured against the Hg/HgO (filled with 1 M KOH), which were then converted to be versus the RHE by the equation U (vs. RHE) = U (vs. Hg/HgO, 1 M KOH) + 0.098 V + 0.059 × pH. The potential of the Hg/HgO electrode relative to the RHE was 0.924 V. The Pt net was used as the counter electrode. Before the test, the cyclic voltammetry (−0.1 to −0.6 V vs. RHE, 0.5 V s^−1^, 40 cycles) was carried out in the Ar-saturated solution. During the test, the acetylene was kept bubbling into the cell. In a typical experiment, the reaction at each potential was carried out for 2 h before analyzing the products. For the analysis with *iR* compensation, a 90% *iR* compensation was used. The *R* for *iR* correction was determined by impedance measurements.

The diameter of the RDE is 5 mm. The surface area of the electrode is 0.196 cm^2^. The surface of glassy carbon was polished by aluminia polishing powder (0.05 μm). Totally, 2 mg Cu MPs was suspended in 0.5 mL ethanol with 10 μL Nafion solution to form a homogeneous ink by ultrasound. Then 25 μL of the ink was drop-casted onto the surface of glassy carbon. The rotation rate is 2500 rpm during the tests.

ECSAs of the Cu MPs catalyst were measured by using the Pb underpotential deposition method in solution with 10 mM Pb(ClO_4_)_2_ and 0.1 M HClO_4_^32,33^. N_2_ was kept bubbling into the system to avoid the effect of O_2_. The 1 × 1 cm^2^ square part of the electrode was immersed in the solution. The cyclic voltammetry test was repeated from −0.375 to −0.005 V vs. Ag/AgCl at 10 mV/s until reproducible voltammograms were obtained. The number of charges consumed for the oxidation of Pb atoms was determined by integrating the anodic shadow area as shown in Supplementary Fig. 8. A conversion factor of 310 μC cm^−2^ was used for Pb oxidation^34^.

### In-situ XANES

XANES spectra were measured at the BL14W1 beamline of the Shanghai Synchrotron Radiation Facility. The specially designed H-type cell was used as the electrolytic cell. The electrode settings and reaction conditions were kept the same as those in the reaction performance tests. In situ XANES spectra was collected at OCP after bubbling Ar or acetylene for 20 min. The gas was kept bubbling into the cell during the measurements. The potential was set at −0.6 V vs. RHE for 10 min before recording the XANES spectra.

### In-situ Raman

Raman spectra were measured with a Xplora confocal microprobe Raman system (HORIBA JobinYvon) equipped with a 50× microscope objective. The working electrode was glassy carbon loaded with Cu MPs. The Hg/HgO electrode (filled with 1 M KOH) and the Pt wire were used as the reference electrode and the counter electrode, respectively. 1 M KOH solution was used as the electrolyte. A laser with a wavelength of 637 nm was used as the excitation source. The power of the laser was about 6 mW. A Si wafer was used to calibrate Raman frequencies. The collection time of each spectrum was over 15 s. Each spectrum was obtained as an average of twice measurements.

### In-situ ATR-FTIR

A Nexus-870 spectrometer was used to measure the ATR-FTIR with an MCT-A detector. The temperature of detector was controlled using liquid nitrogen. The basal plane of a hemicylindrical Si prism beveled at 60° was deposited on Au film using a chemical method. The catalyst ink was dripped onto the Au film polished by an electrochemical way. 0.1 M KOH solution was used as the electrolyte. The electrode settings and reaction conditions were kept the same as those in the reaction performance tests. The acetylene was constantly introduced into the cell during the test. The reference spectra were collected at OCP.

### Products analysis

The gas-phase products were analyzed by using gas chromatography with Ar as the carrier gas. The GC-2060 was equipped with an Al_2_O_3_/Na_2_SO_4_ column (Kromat 50 m × 0.53 mm × 20.00 μm) on a flame ionization detector which can detect hydrocarbon products. The TDX column was equipped in the GC with a thermal conductivity detector which can detect H_2_. The chromatograph was equipped with a ten-port valve and sample injection loop. During the experiment, the ten-port valve is periodically switched between Position A and Position B (Supplementary Fig. 33). In Position A, the outgoing gas passes through the sample loops and then is vented out. When the ten-ports valve is switched to Position B, the carrier gas brings the sample gas in the sample loops into the chromatography (two columns for TCD and FID, respectively) for analyzing the products. This makes sure that the same volume of gas is injected into the chromatogram in each analysis. The GC measurements were carried out after running the reaction for 2 h.

A typical GC spectrum is shown in Supplementary Fig. 34. The Faradaic efficiency is calculated using the following equation.

2 $${{{{{\rm{FE}}}}}}_{({{{{{\rm{product}}}}}})}=\frac{{S}_{({{{{{\rm{product}}}}}})}}{a}\times v\times t\times \frac{n{{{{{\rm{FP}}}}}}_{0}}{{{{{\rm{RT}}}}}}\times \frac{1}{Q}$$

where, *S*_(product)_ is the area of products on the GC spectrum, *a* is the percentage conversion factor based on standard gas, which converts the peak area to the volume percentage of a single product. *v* is the gas flow rate, *t* is the reaction time, *n* is the moles of charges transfer from reactants to products per mole, *F* is Faraday’s constant 96,485 C mol^−1^. *Q* is the total number of charges applied into the system

*P*_0_ = 101,325 Pa, *R* = 8.314 J mol^−1^ K^−1^, and *T* = 298.15 K.

For the experiments, *v* = 4.8 mL min^−1^ = 0.8 × 10^−7^ m^3^ s^−1^; *t* = 2 h = 7200 s. In the experiments with tandemly connected cells, *v* is adjusted to 2.4 mL min^−1^.

The ${j}_{{{{{\rm{C}}}}}_{2}{{{{\rm{H}}}}}_{2}}$ and ${j}_{{{{{\rm{C}}}}}_{2}{{{{\rm{H}}}}}_{4}}$ are calculated using the following equations:

3 $${j}_{{{{{\rm{C}}}}}_{2}{{{{\rm{H}}}}}_{2}}=\mathop{\sum}\limits_{{{{{\rm{product}}}}}}{{{{{\rm{FE}}}}}}_{({{{{\rm{product}}}}})}\times j$$

4 $${j}_{{{{{\rm{C}}}}}_{2}{{{{\rm{H}}}}}_{4}}={{{{{\rm{FE}}}}}}_{{{{{\rm{C}}}}}_{2}{H}_{4}}\times j$$

where FE_(product)_ is the Faradaic efficiency of a specific product of the electrochemical acetylene hydrogenation, ${{{{{\rm{FE}}}}}}_{{{{{\rm{C}}}}}_{2}{H}_{4}}$ is the Faradaic efficiency of ethylene formation, and *j* is the overall current density.

The acetylene reaction rate per unit area (*r*_ace_) was calculated from the formation rates of products using the following equation:

5 $${r}_{{{{{\rm{ace}}}}}}={\sum }_{{{{{\rm{product}}}}}}\frac{{n}_{({{{{{\rm{ace/product}}}}}})}\times \frac{j\times {{{{{\rm{FE}}}}}}_{({{{{{\rm{product}}}}}})}\times t}{{z}_{({{{{{\rm{product}}}}}})}\times F}}{t}={\sum }_{{{{{\rm{product}}}}}}\frac{{n}_{({{{{{\rm{ace/product}}}}}})}\times j\times F{E}_{({{{{{\rm{product}}}}}})}}{{z}_{({{{{{\rm{product}}}}}})}\,\times \,F}$$

where *n*_(ace/product)_ is the moles of acetylene consumed to produce one mole of a specific product, *j* is the overall current density, *FE*_(product)_ is the Faradaic efficiency of a specific product, *t* is reaction time (2 h), *z*_(product)_ is the moles of transferred charges for producing one mole of a specific product, *F* is the Faraday’s constant 96,485 C mol^−1^.

Meanwhile, none of liquid phase product was detected by nuclear magnetic resonance (Supplementary Fig. 35).

### Computational details

First-principles periodic calculations were carried out with the Vienna Ab initio Simulation Package based on DFT methods^35,36^. The Perdew–Burke–Ernzerhof generalized gradient approach^37,38^ was used to define the exchange-correlation potential. The interaction between the atomic cores and electrons was described using the projector augmented wave potential^39,40^. The surface brillouin zone was sampled at 1 × 1 × 1 k-point. A 400 eV energy cutoff and the force convergence criterion for each relaxed atom below 0.02 eV/Å were used. Zero-damping DFT-D3 method of Grimme was used for the Van der Waals correction^41,42^. To determine the kinetic barriers for the electron-coupled proton transfer hydrogenation steps, we introduced a water network model in the system above the surface specie to simulate the transfer of a proton from one of the water molecules to the targeted surface specie. TS searches were conducted at the same theoretical level using Fixed Bond Length method as implemented in the Atomic Simulation Environment by determining the highest energy point along the potential energy surface^43^, and were checked with imaginary vibrational frequencies. The Cu surfaces were built as a four-layer slab in a 6 × 6 supercell with the bottom two layers being fixed during optimization. A vacuum thickness larger than 15 Å was used to separate the surface from its periodic image in the direction perpendicular to the surface.

The adsorption energies (Δ*E*_ads_) of the adsorbed molecular species were calculated using the equation below

6 $${\varDelta E}_{{{{{\rm{ads}}}}}}{=E}_{\left({{{{\rm{adsorbate}}}}}+{surface}\right)}-{E}_{\left({{{{\rm{surface}}}}}\right)}-{E}_{\left({{{{\rm{adsorbate}}}}}\right)}$$

where *E*_(adsorbate+surface)_, *E*_(surface)_, and *E*_(adsorbate)_ are the energies of the optimized surface containing the adsorbate, the optimized clean surface and the optimized adsorbate in the gas phase, respectively. In addition, free energy corrections were considered to obtain the adsorption free energy (Δ*G*_ads_) and activation free energy (Δ*G*_act_) using the expression as follows:

7 $${\varDelta G}_{{{{{\rm{ads}}}}}}={\varDelta E}_{{{{{\rm{ads}}}}}}+{\varDelta E}_{{{{{\rm{ZPE}}}}}}+{\varDelta U}_{{{{{\rm{vib}}}}}}-T\varDelta S$$

8 $${\varDelta G}_{{{{{\rm{act}}}}}}={\varDelta E}_{{{{{\rm{act}}}}}}+{\varDelta E}_{{{{{\rm{ZPE}}}}}}+{\varDelta U}_{{{{{\rm{vib}}}}}}$$

where Δ*E*_ads_ is the 0 K adsorption energy, Δ*E*_act_ is the 0 K activation energy, Δ*E*_ZPE_ represents the change in the zero-point vibrational energy, Δ*U*_vib_ represents the change in the vibrational part of the internal energy, and Δ*S* represents the change in the entropy, for the adsorption of the adsorbates or between the TSs and initial states at room temperature (*T* = 25 °C). The calculated vibrational corrections and frequencies are shown in Supplementary Tables 5–17. The surface coverage in ML was defined as

9 $${\theta }={N}_{{{{{\rm{C}}}}}}/{{{{{\rm{NS}}}}}}_{{{{{\rm{Cu}}}}}}$$

where *N*_C_ and NS_Cu_ represent the total number of carbon atoms of the surface-adsorbed C_2_H_2_ and the number of surface Cu atoms, respectively.

For an electrochemical reaction

10 $${{{{{\rm{M}}}}}}^{* }+{{{{{\rm{H}}}}}}_{({{{{\rm{aq}}}}})}^{+}+{{{{{\rm{e}}}}}}^{-}\to {{{{{\rm{MH}}}}}}^{* }$$

the potential dependent reaction free energy change referenced to a RHE using the computational hydrogen electrode approach^44^, is calculated as shown below

11 $$\triangle G={G}_{{{{{{\rm{MH}}}}}}^{* }}-{G}_{{{{{\rm{M}}}}}^{* }}-\frac{1}{2}{G}_{{{{{\rm{H}}}}}_{2}(g)}+{eU}$$

where *G*_MH*_, *G*_M*_ and $G_{{{{{{\mathrm{H}}}}}}_{2}({{{{{\mathrm{g}}}}}})}$ represent the Gibbs free energies for the product, reactant and the gas phase H_2_, respectively, at room temperature, and *U* is the potential relative to the RHE. To determine the energy barrier at −0.6 V vs. RHE, we used the method proposed by Nie et al.^45^, as shown in the following expression

12 $${E}_{{{{{\rm{act}}}}}}(U)={E}_{{{{{\rm{act}}}}}}^{0}({U}^{0})+{\beta }{{{\hbox{'}}}}(U-{U}^{0})$$

where ${E}_{{{{{\rm{act}}}}}}^{0}$ is the activation energy barrier determined by standard DFT approach, *U*^0^ is the equilibrium potential, and it is calculated using Eq. (11) with respect to our system, by determining the potential at which the reaction free energy is zero (Supplementary Table 18). *U* is the electrode potential relative to the RHE, and *β*′ is the effective symmetry factor, which is assumed to be 0.5 for our system.

To estimate the charge transfer after adsorption, we use the Bader’s approach as numerically applied by Henklemann’s group^46,47^. To understand the charge redistribution after C_2_H_2_ adsorption on Cu surfaces, we plotted the charge density difference by subtracting the densities of C_2_H_2_ and Cu surface from the density of C_2_H_2_/Cu as expressed below

13 $$\triangle \rho =\rho ({{{{\rm{C}}}}}_{2}{H}_{2}/{Cu})-\rho ({C}_{2}{H}_{2})-\rho \left({Cu}\right)$$

where *ρ*(C_2_H_2_/Cu), *ρ*(C_2_H_2_), and *ρ*(Cu) are the charge densities of the C_2_H_2_/Cu system, and the corresponding parts of C_2_H_2_ and Cu, respectively. By using the same atom positions as those in C_2_H_2_/Cu, the charge densities of the latter two systems (C_2_H_2_ and Cu) were obtained.

## Supplementary information

Supplementary Information

## Data Availability

The data supporting the findings of this study are available within the article and its Supplementary Information. The optimized atomic positions of the reaction intermediates and transition states are available in the figshare repository (10.6084/m9.figshare.14184587). Source data are provided with this paper. Additional data are available from the corresponding authors on reasonable request.

## Source data

Source Data
