# Supplementary material for: Highly efficient ethylene production via electrocatalytic hydrogenation of acetylene under mild conditions
Source: Nat Commun. 2021 Dec 6;12:7072. doi: 10.1038/s41467-021-27372-8 (PMC8648715; doi:10.1038/s41467-021-27372-8)
Supplement: Supplementary file 1 — Supplementary Information [file 41467_2021_27372_MOESM1_ESM.pdf]

Supplementary Information for

Highly efficient ethylene production via electrocatalytic hydrogenation of acetylene under mild conditions

Suheng Wang[†1,2], Kelechi Uwakwe[†2,3], Liang Yu[2,3*], Jinyu Ye[1], Yuezhou Zhu[1], Jingting Hu[1,2], Ruixue Chen[1,2], Zheng Zhang[1,2], Zhiyou Zhou[1], Jianfeng Li[1], Zhaoxiong Xie[1] and Dehui Deng[1,2,3*]

[1]State Key Laboratory for Physical Chemistry of Solid Surfaces, Collaborative Innovation Center of Chemistry for Energy Materials, College of Chemistry and Chemical Engineering, Xiamen University, Xiamen 361005, China.

[2] State Key Laboratory of Catalysis, Collaborative Innovation Center of Chemistry for Energy Materials, Dalian Institute of Chemical Physics, Chinese Academy of Sciences, Dalian 116023, China.

[3]University of Chinese Academy of Sciences, Beijing 100049, China.

[†] These authors contributed equally.

Correspondence and requests for materials should be addressed to L.Y. and D.D. (email: lyu@dicp.ac.cn; dhdeng@dicp.ac.cn)

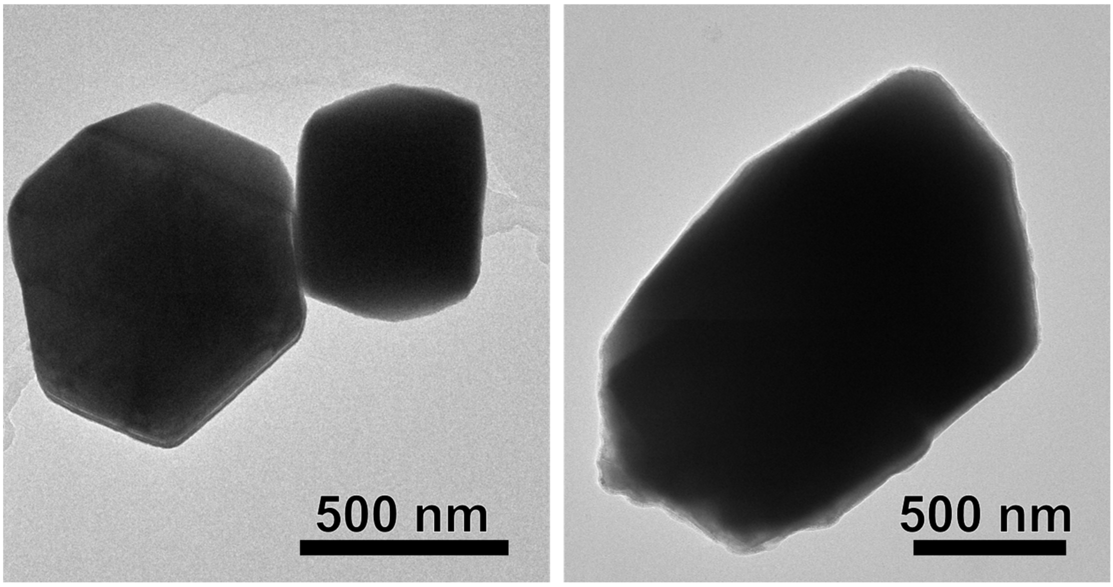

Supplementary Figure 1 | Morphology characterizations of Cu MPs. The TEM images of Cu MPs.

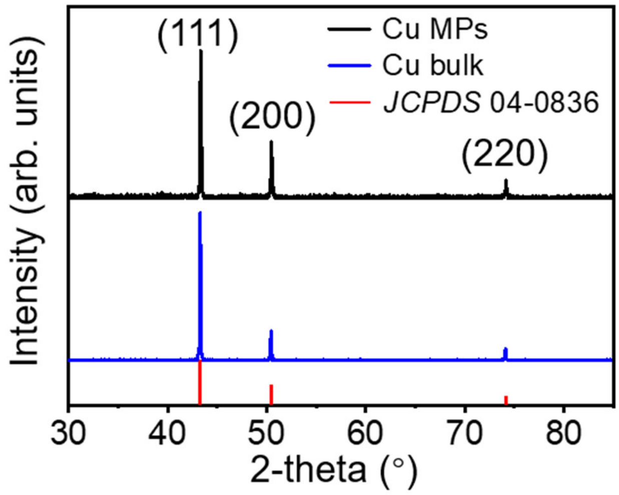

Supplementary Figure 2 | XRD characterizations of the crystal structure of Cu MPs. The XRD patterns of the Cu microparticles (MPs) and commercial Cu bulk with peak intensities normalized based on the (111) peak. The size of the Cu bulk is 10 × 10 × 1 mm. The (200) and (220) facets are equivalent with the (100) and (110) facets on the surface atomic structure and periodicity. The absence of the (110) and (100) facets in the XRD patterns is because that they do not meet the allowed reflection conditions for the (*h*,*k*,*l*) miller indices of the face-centered cubic (FCC) structure, which require the indices to be either all odd or all even[1]. Source data are provided with this paper.

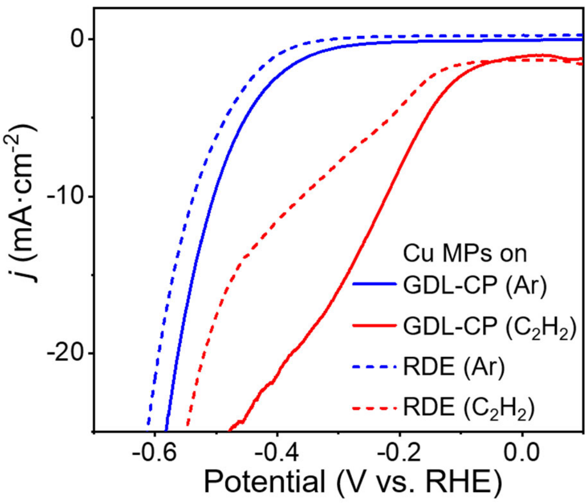

Supplementary Figure 3 | The voltammetric characterizations of Cu MPs under different gas saturated solutions on different electrodes. Linear sweep voltammetric curves with *i*R compensation on Cu/GDL-CP and Cu/RDE in Ar-saturated and C_2H_2-saturated solution. For the Cu/GDL-CP, 4 mg Cu MPs is loaded on 1 cm^2 (1 × 1 cm) GDL-CP. For the Cu/RDE, 0.1 mg Cu MPs is loaded on the rotating disk electrode with 5 mm diameter (0.196 cm^2). The rotation rate is 2500 rpm. The scanning rate is 10 mV s^{-1}. The larger difference in the LSV curves on the two different electrodes in the C_2H_2-saturated solution should be due to the effect of the gas diffusion layer, which promotes the E-HAE reaction on the Cu/GDL-CP electrode. Source data are provided with this paper.

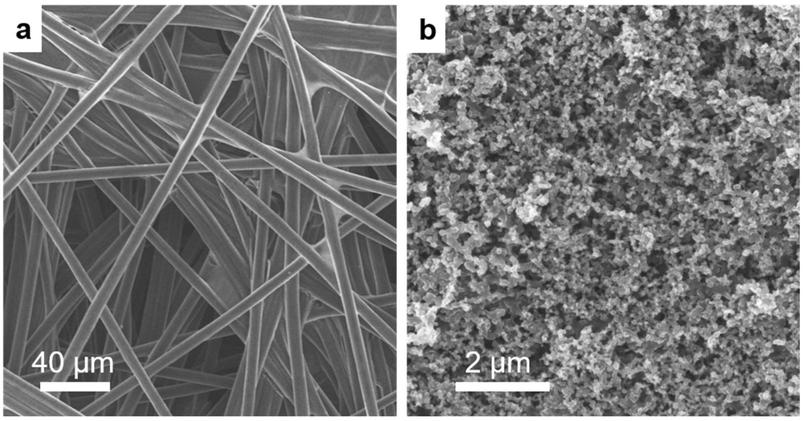

Supplementary Figure 4 | SEM characterizations of different carbon papers. SEM images of carbon paper (**a**) and carbon paper with gas diffusion layer (**b**).

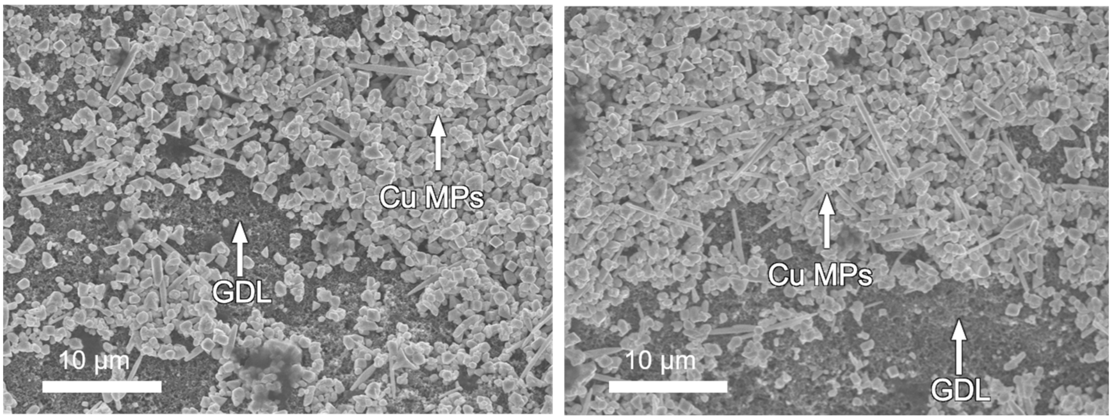

Supplementary Figure 5 | SEM characterizations of the Cu/GDL-CP. The SEM images of the Cu/GDL-CP.

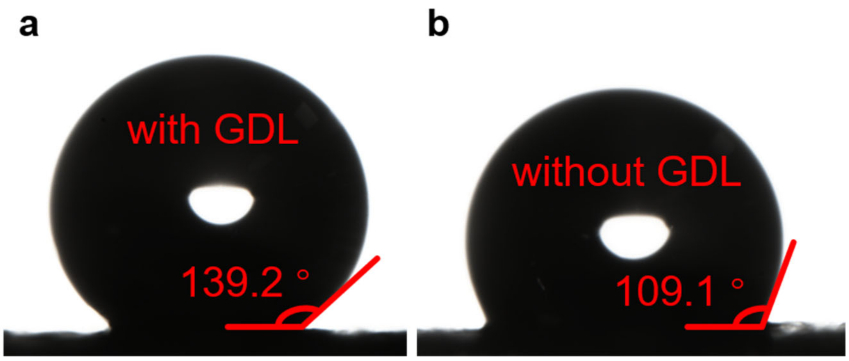

Supplementary Figure 6 | The contact angle test of the Cu/GDL-CP and Cu/CP electrodes. The comparison in the contact angle of an aqueous droplet on the surface of the Cu/GDL-CP (**a**) and Cu/CP (**b**) electrodes.

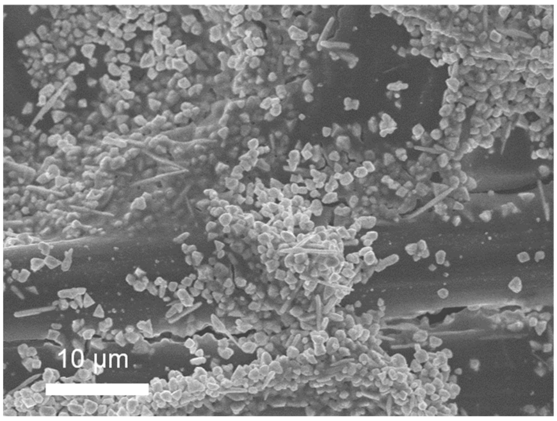

Supplementary Figure 7 | SEM characterization of the Cu/CP electrode. The SEM image of the Cu/CP.

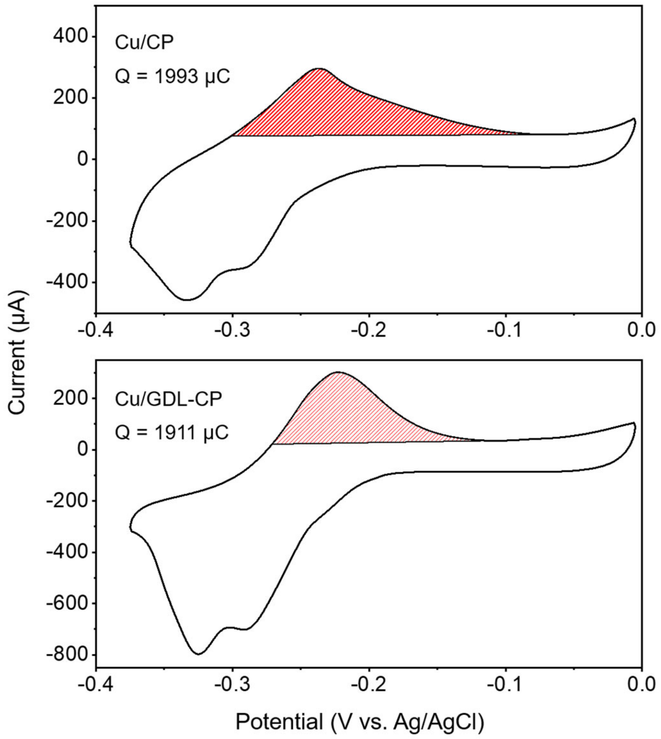

Supplementary Figure 8 | Measurements of the electrochemical active surface areas of the Cu/CP and Cu/GDL-CP electrodes. Pb UPD and stripping cyclic voltammetry curves over the two electrodes in the solution of 10 mM $Pb(ClO_4)_2$ and 0.1 M $HClO_4$ at 25 °C. The loading of Cu MPs on the CP in all cases is 4 mg. The scan speed was 10 mV/s. The two peaks at -0.29 V and -0.32 V should be assigned to the deposition of Pb on the Cu(111) and Cu(100) surfaces[2,3,4], respectively, which are the dominant surfaces exposed. Source data are provided with this paper.

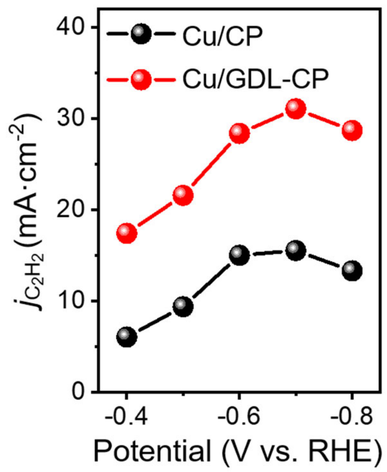

Supplementary Figure 9 | The geometric current density of acetylene consumption ($j_{C_2H_2}$) over the Cu/GDL-CP and Cu/CP electrodes. The test was carried out in 1 M KOH solution at 25 ºC. Source data are provided with this paper.

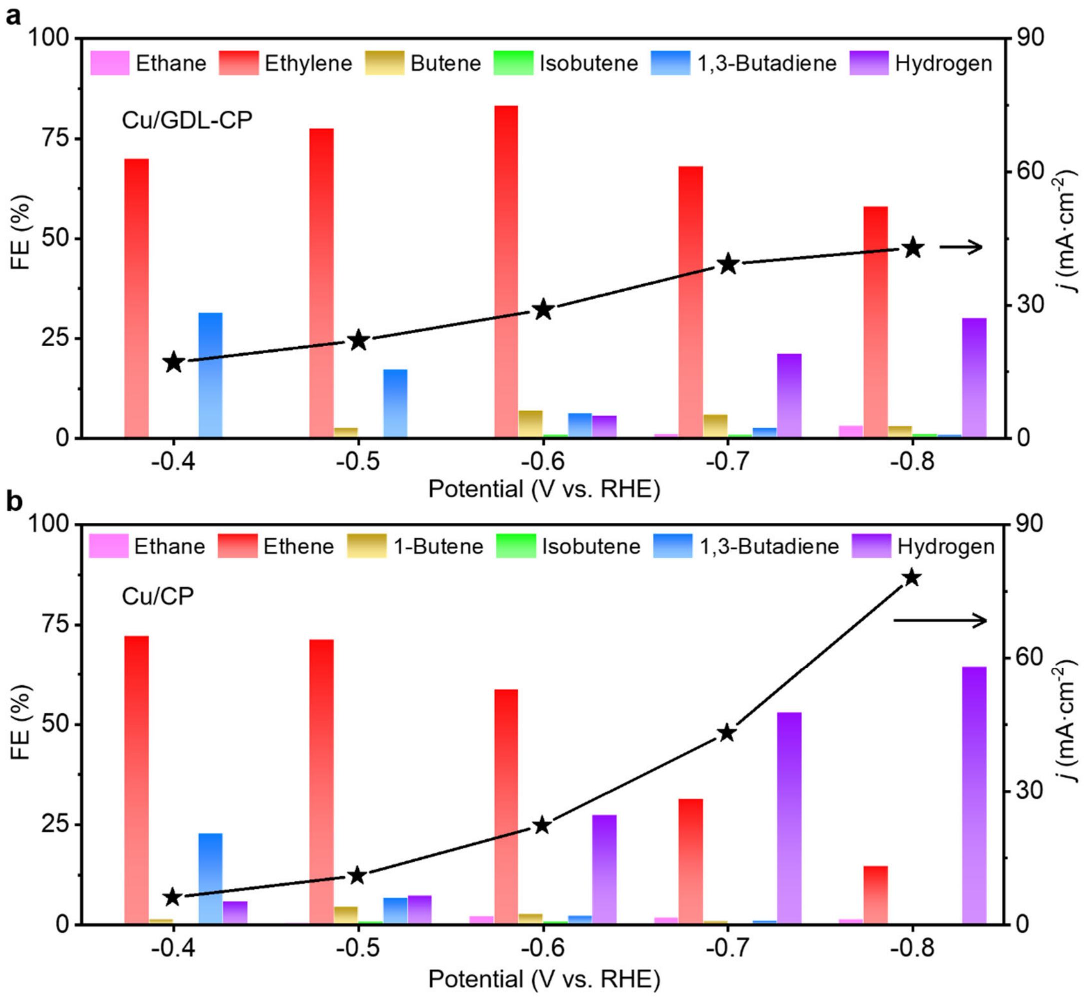

Supplementary Figure 10 | The electrochemical performance of the Cu/GDL-CP and Cu/CP electrodes. FEs of the E-HAE products and the current densities at different potentials over the Cu/GDL-CP (**a**) and Cu/CP electrodes (**b**). Source data are provided with this paper.

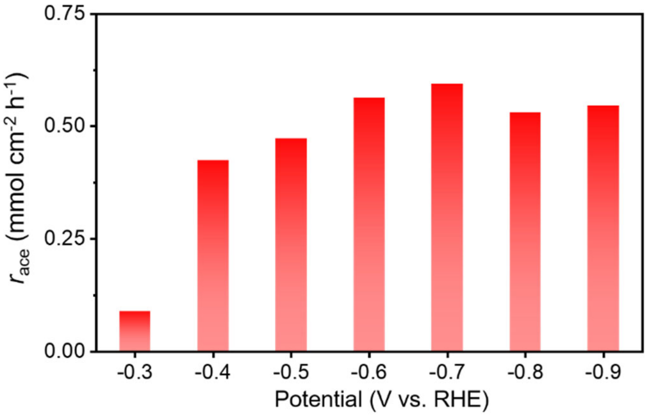

Supplementary Figure 11 | The E-HAE performance of the Cu/GDL-CP electrode. The rate of acetylene consumption (r_{ace}) at different potentials. Source data are provided with this paper.

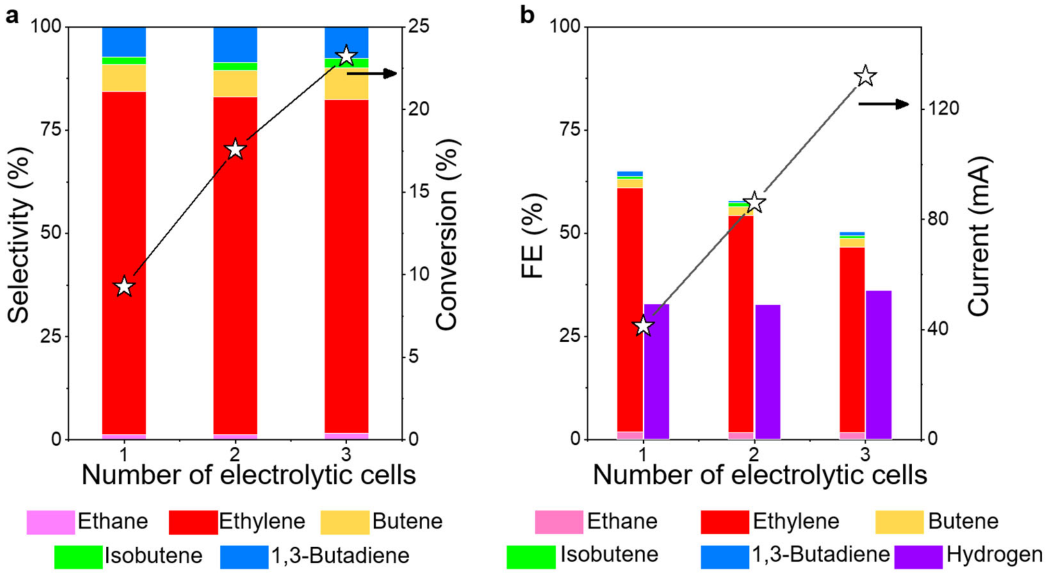

Supplementary Figure 12 | The E-HAE performance of tandemly connected electrolytic cells. The conversion and selectivity (**a**), Faradaic efficiency and current densities (**b**) of E-HAE at -0.7 V vs. RHE in tandemly-connected electrolytic cells. The flow rate was adjusted to 2.4 mL/min. The other reaction conditions of each electrolytic cell were kept the same. The acetylene reaction rate was 0.45 mmol cm^{-2} h^{-1} in the three-cell system. Source data are provided with this paper.

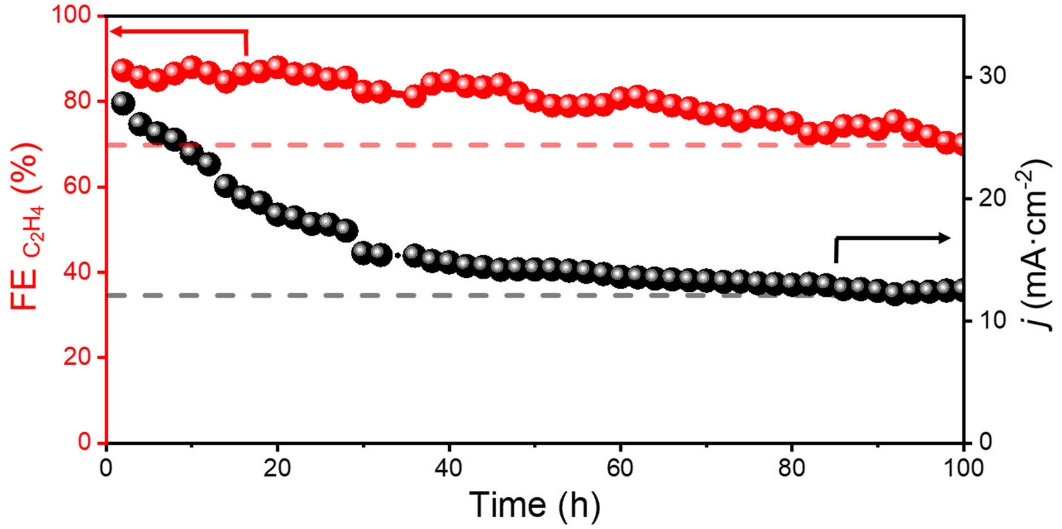

Supplementary Figure 13 | The E-HAE stability performance of Cu MPs catalyst. The test was carried out at -0.6 V vs. RHE in 1 M KOH solution at 25 ºC. Source data are provided with this paper.

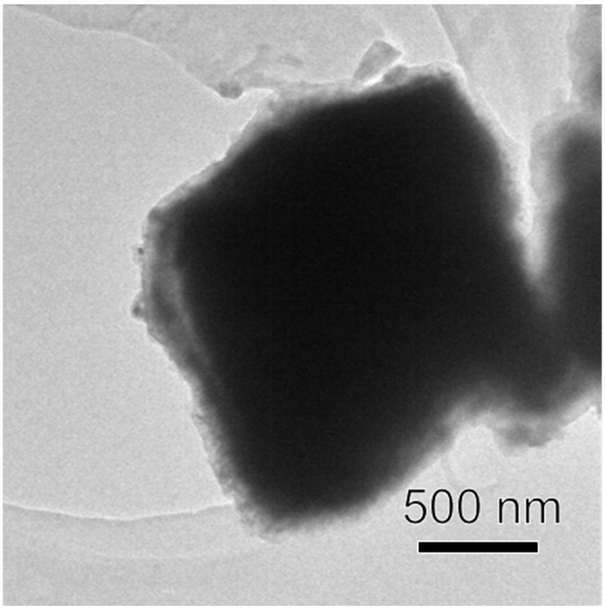

Supplementary Figure 14 | TEM characterization of Cu MPs after stability test. The TEM image of the Cu catalyst after the 100-hour stability test. The stability test was carried out in 1 M KOH solution at 25 ºC and -0.6 V vs. RHE. Source data are provided with this paper.

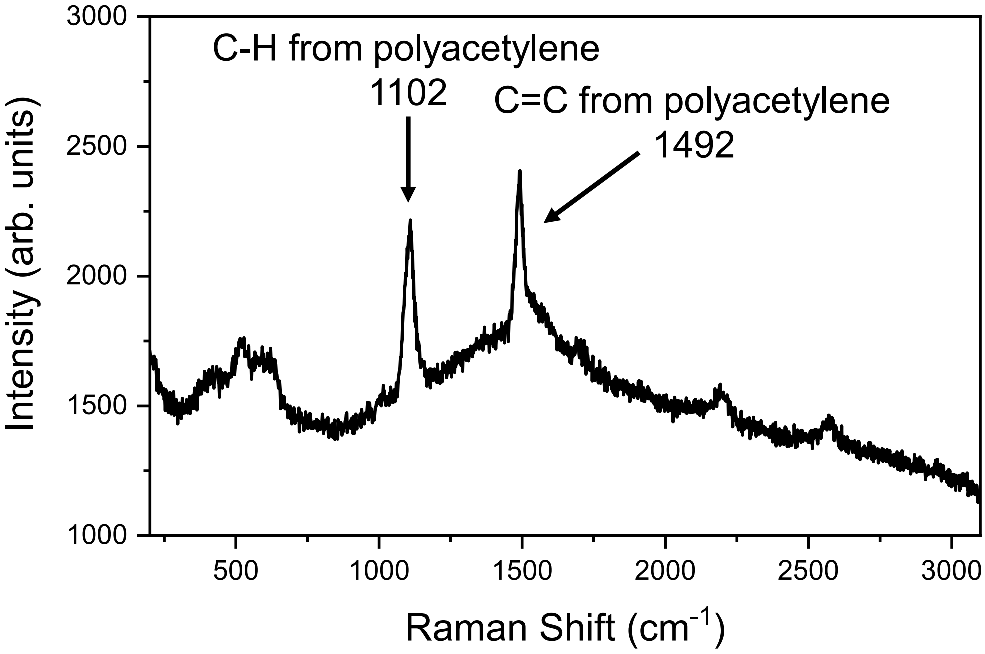

Supplementary Figure 15 | Characterization of the Cu MPs after the stability test. The Raman spectrum of the Cu catalyst after the 100-hour stability test. The stability test was carried out in 1 M KOH solution at 25 ºC and -0.6 V vs. RHE. Source data are provided with this paper.

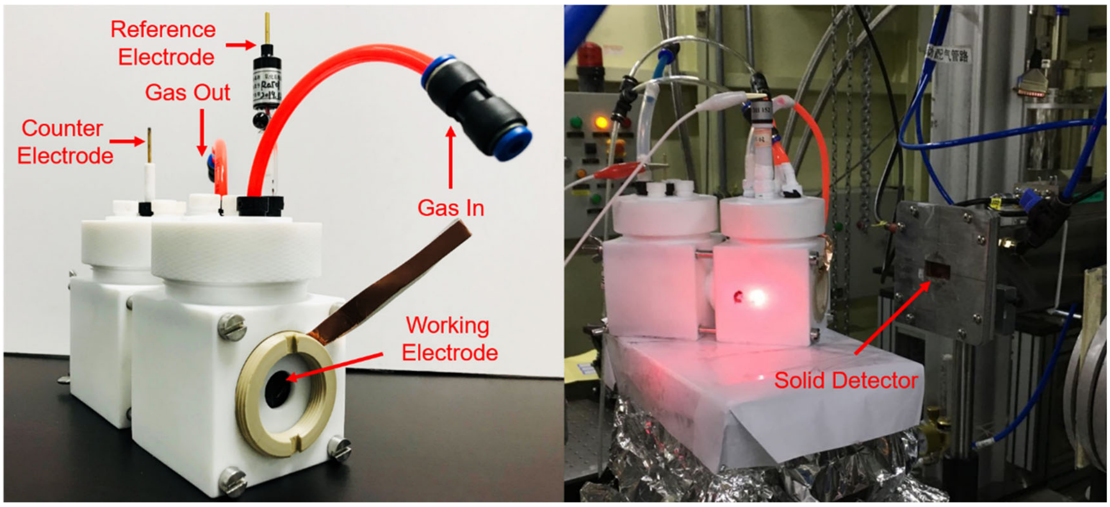

Supplementary Figure 16 | Photos of the electrolytic cell for in-situ XANES characterizations. The test was carried out at the BL14W1 beamline of the Shanghai Synchrotron Radiation.

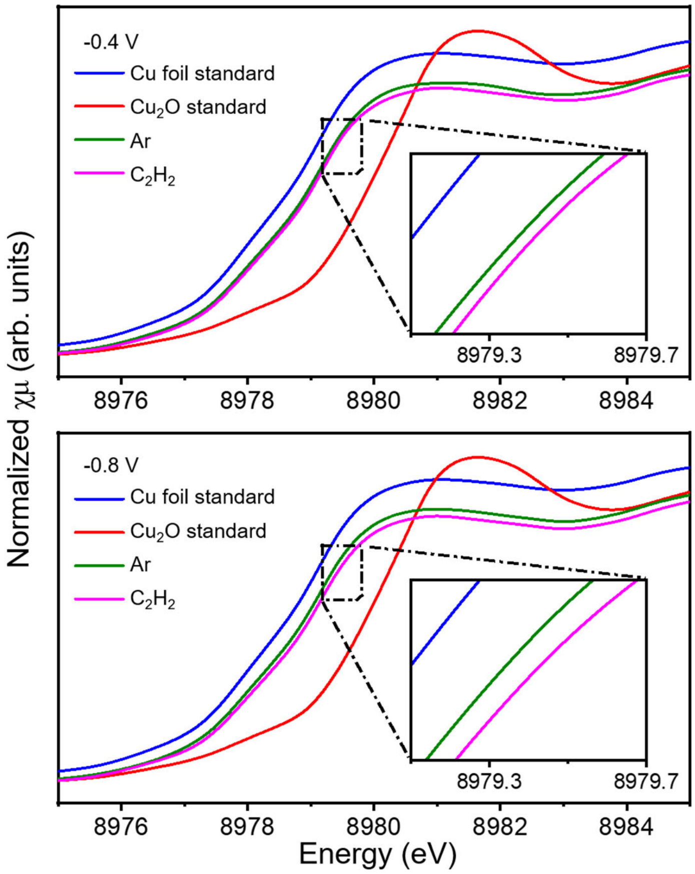

Supplementary Figure 17 | In-situ XANES characterizations of the Cu MPs catalyst. The in-situ Cu K-edge XANES of the catalyst in Ar-saturated and C_2H_2-saturated solution at -0.4 V and -0.8 V vs. RHE. Source data are provided with this paper.

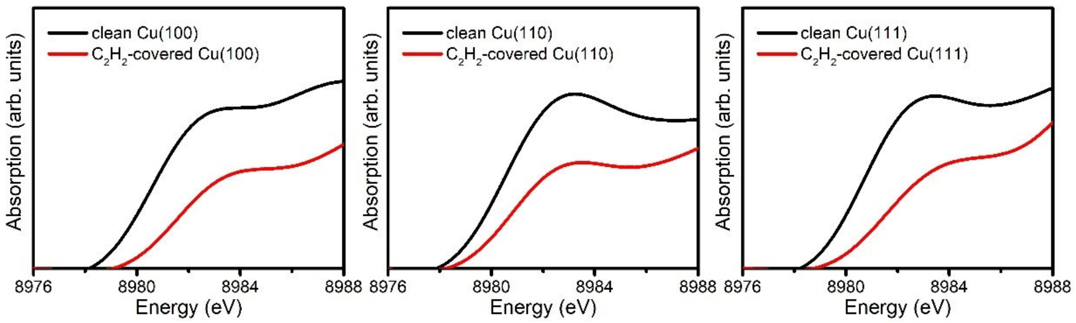

Supplementary Figure 18 | DFT Simulation of the XANES results. DFT-simulated K-edge XANES of the surface Cu atoms of clean and C_2H_2-covered Cu(100), Cu(110), and Cu(111), respectively.

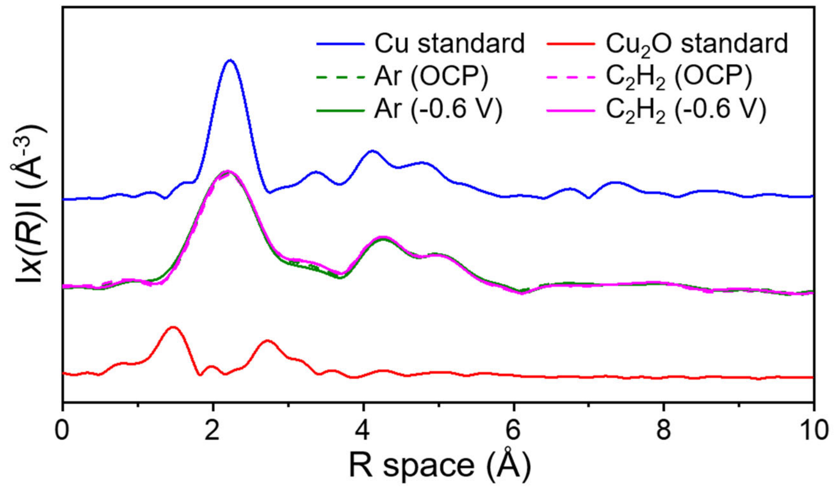

Supplementary Figure 19 | In-situ EXAFS characterizations of the Cu MPs catalyst. Fourier trnsformed in-situ EXAFS spectra in the R space for the Cu MPs catalyst in Ar-saturated and C_2H_2-saturated solution at OCP and at -0.6 V vs. RHE, respectively, in comparison with those for the Cu foil and Cu_2O standard samples. The spectra at -0.6 V vs. RHE were measured after running the reaction for 4 hours. Source data are provided with this paper.

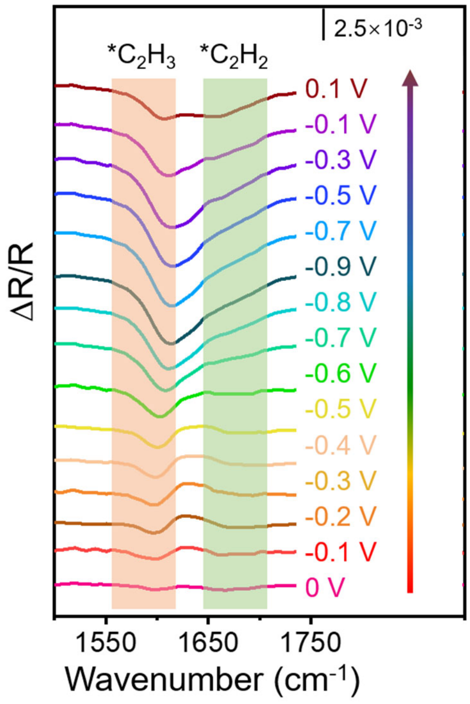

Supplementary Figure 20 | The in-situ ATR-FTIR spectra of the Cu MPs during the E-HAE. The spectra were measured at potentials changing from 0 V to -0.9 V and then back to 0.1 V. All the potentials are referenced to the RHE. The in-situ ATR-FTIR tests were carried out in 0.1 M KOH solution at 25 ºC. Source data are provided with this paper.

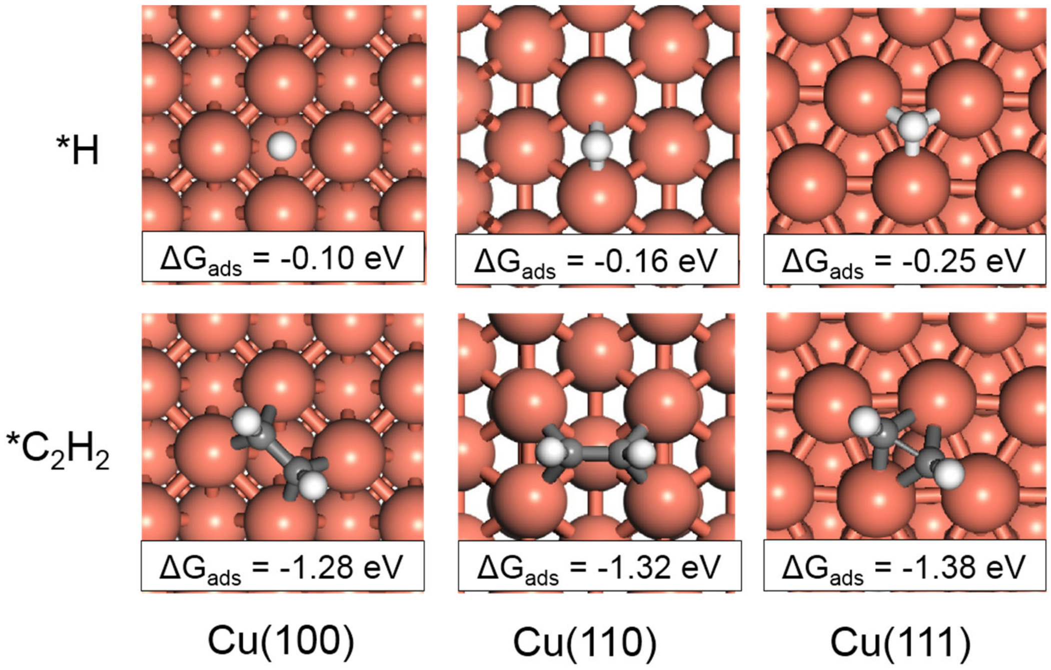

Supplementary Figure 21 | The adsorption of *H and *C_2H_2 on different Cu facets. Most stable adsorption configurations and adsorption free energies of *H and *C_2H_2 on the Cu(100), Cu(110) and Cu(111) clean surfaces (H in white, C in gray, and Cu in dark orange).

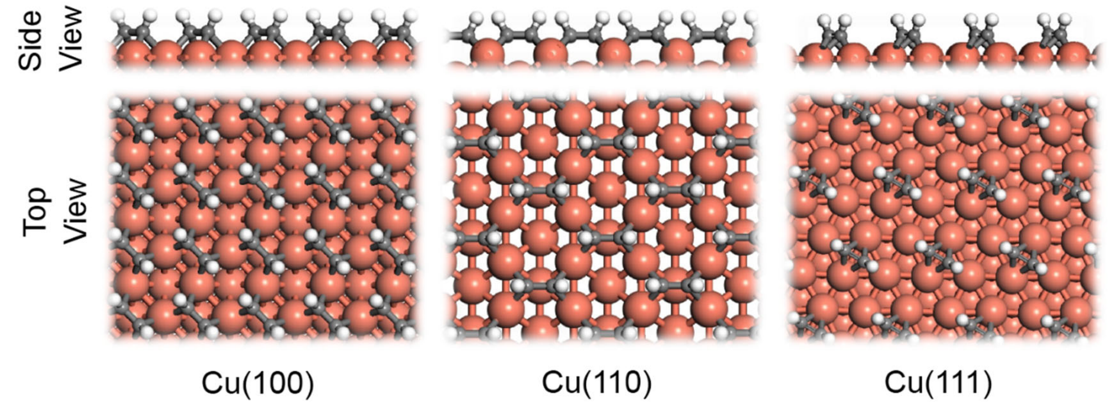

Supplementary Figure 22 | The structures of C_2H_2-covered Cu surfaces. The Cu(100) and Cu(110) are covered with 1.0 ML C_2H_2 and Cu(111) with 0.5 ML C_2H_2 (H in white, C in gray, and Cu in dark orange). The surface coverage in ML was defined as $\theta = N_C/NS_{Cu}$, where N_C and NS_{Cu} represent the total number of carbon atoms of the surface-adsorbed C_2H_2 and the number of surface Cu atoms, respectively.

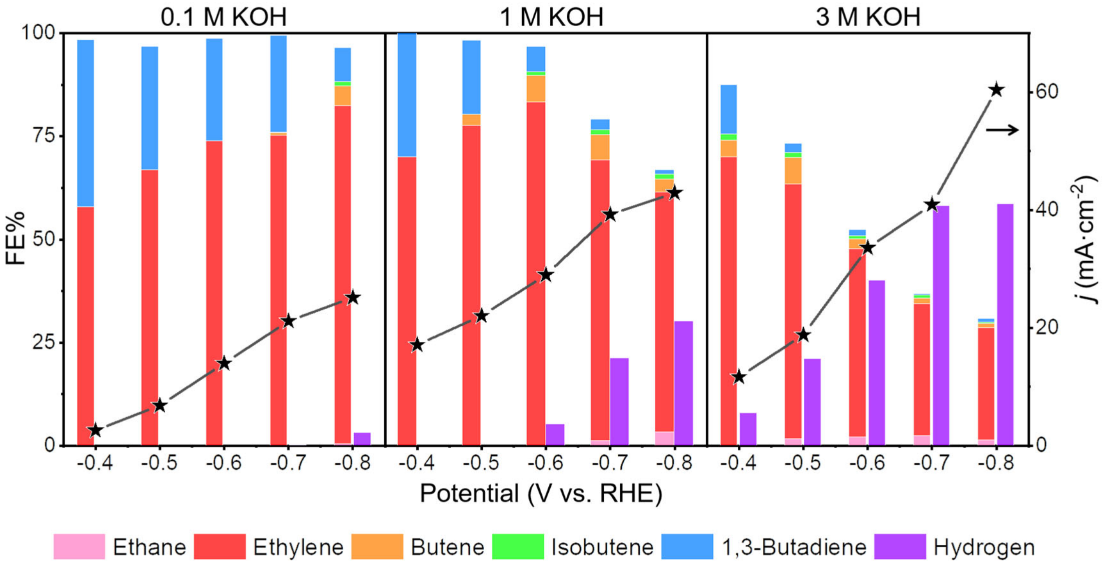

Supplementary Figure 23 | The E-HAE performance of the Cu/GDL-CP under different KOH concentrations. FE of the E-HAE products at different potentials in alkaline solutions with different KOH concentrations at 25 °C. The working electrode is Cu/GDL-CP with Cu MPs loading amount of 4 mg. Considering that the current density (*j*) is also significantly affected by the ionic strength in the solution, thus it is not used to analyze the activity of the catalyst. Source data are provided with this paper.

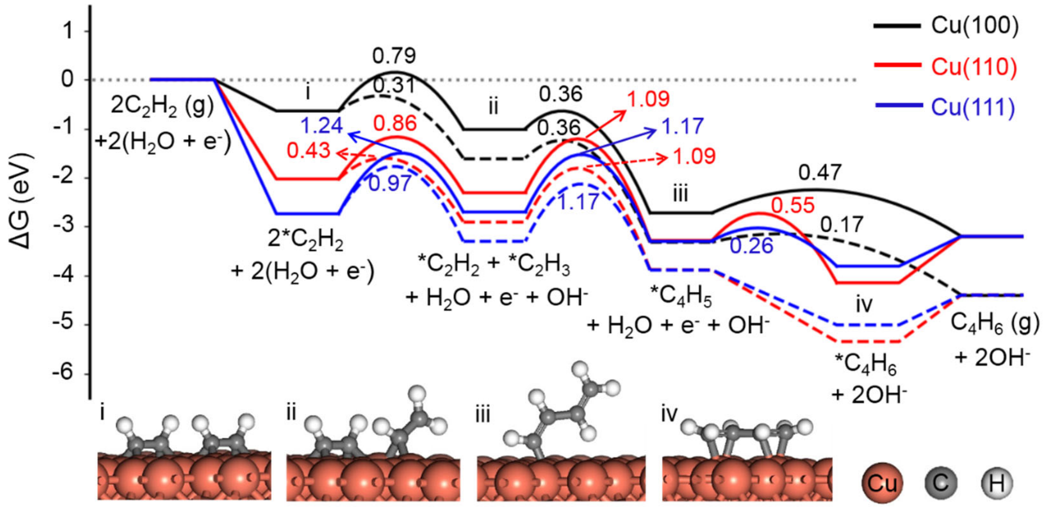

Supplementary Figure 24 | Free energy diagram for the formation of C_4H_6 (First Pathway) on the C_2H_2-covered Cu(100), Cu(110) and Cu(111) surfaces at 0 V and -0.6 V vs. RHE, 25 °C. The solid lines represent the potential energy surface at 0 V, while the dashed lines represent the potential energy surface at -0.6 V. For a clearer description of the reaction states only the species concerned are shown in the inserted screenshots, the neighboring adsorbed species are not shown for simplicity.

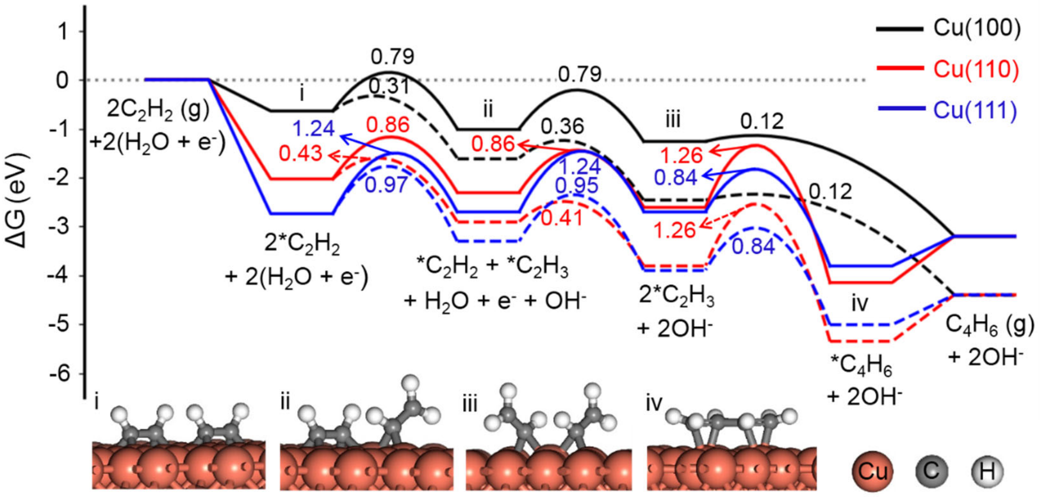

Supplementary Figure 25 | Free energy diagram for the formation of C_4H_6 (Second Pathway) on the C_2H_2-covered Cu(100), Cu(110) and Cu(111) covered surfaces at 0 V and -0.6 V vs. RHE, 25 °C. The solid lines represent the potential energy surface at 0 V, while the dashed lines represent the potential energy surface at -0.6 V. For a clearer description of the reaction states only the species concerned are shown in the inserted screenshots, the neighboring adsorbed species are not shown for simplicity.

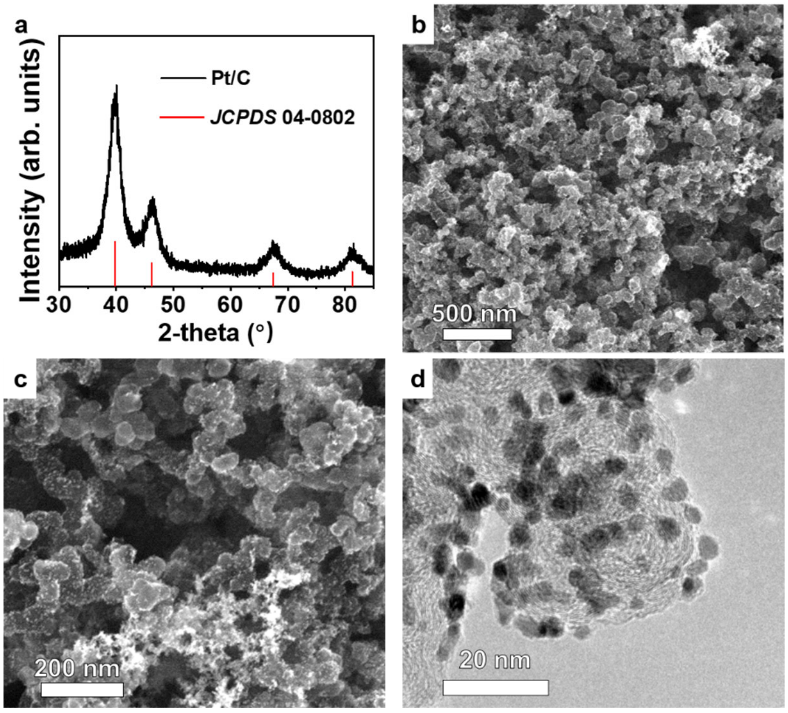

Supplementary Figure 26 | The structural characterizations of Pt/C. The XRD pattern (a), SEM images (b-c), and TEM image (d) of Pt/C. Source data are provided with this paper.

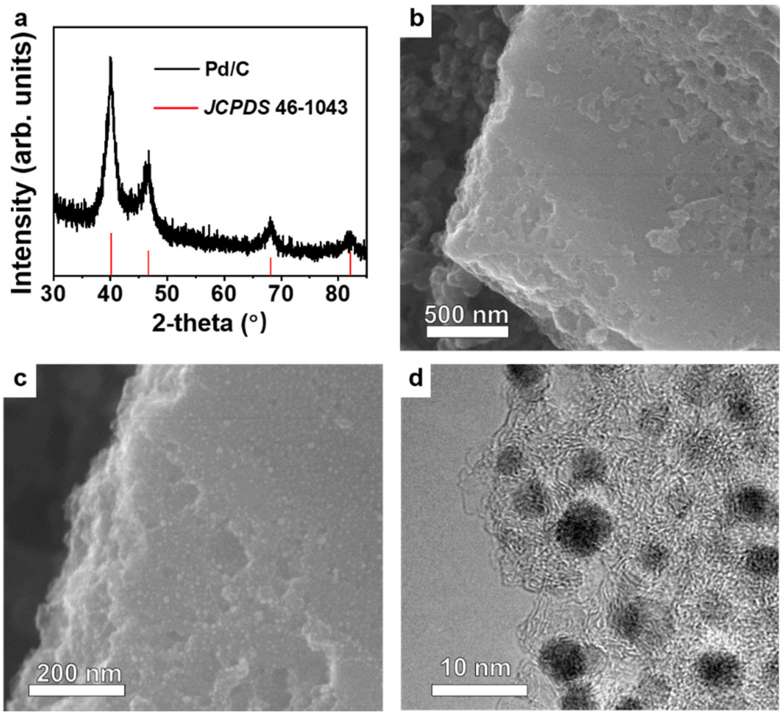

Supplementary Figure 27 | The structural characterizations of Pd/C. The XRD pattern (a), SEM images (b-c), and TEM image (d) of Pd/C. Source data are provided with this paper.

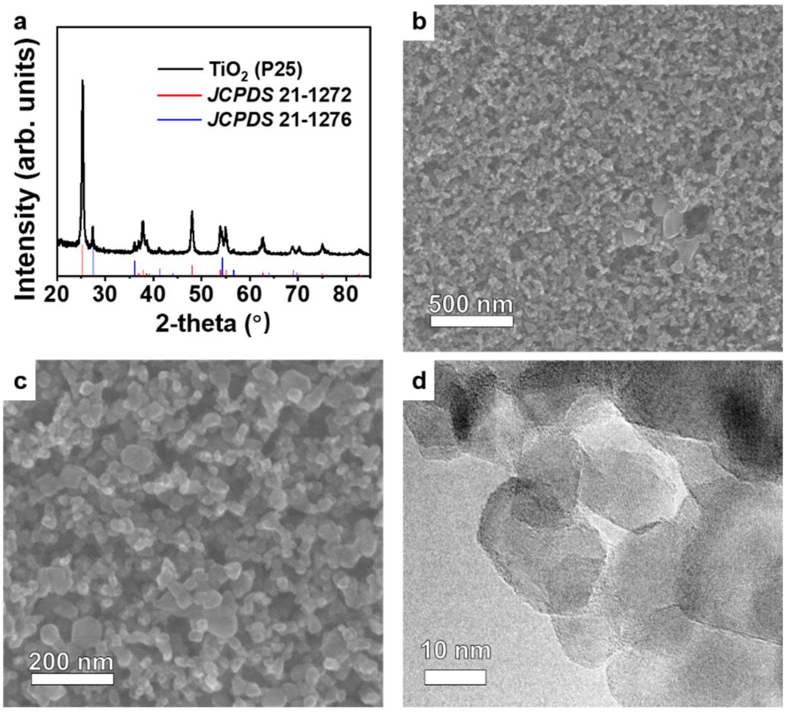

Supplementary Figure 28 | The structural characterizations of TiO_2. The XRD pattern (a), SEM images (b-c), and TEM image (d) of TiO_2 (P25). Source data are provided with this paper.

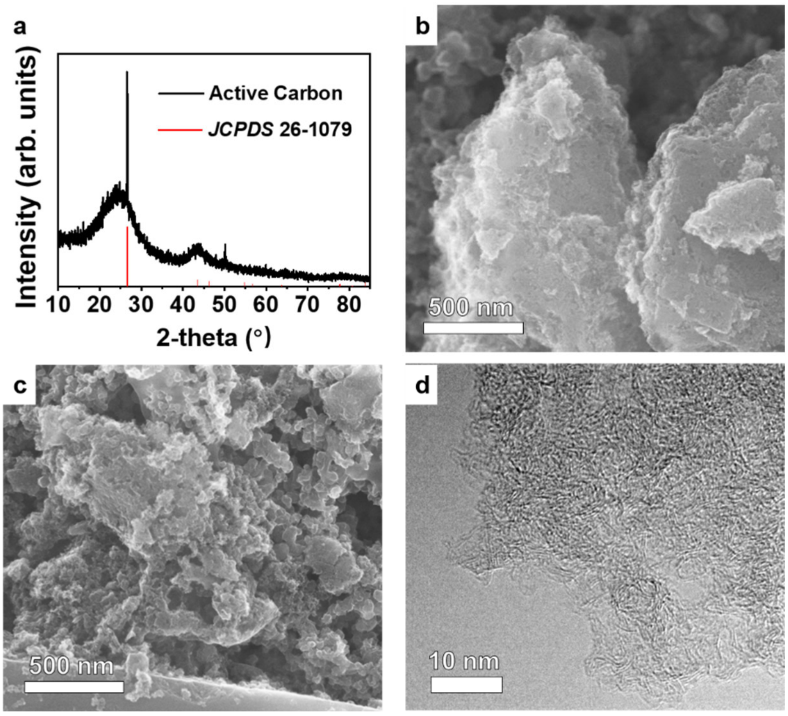

Supplementary Figure 29 | The structural characterizations of active carbon. The XRD pattern (a), SEM images (b-c), and TEM image (d) of active carbon. Source data are provided with this paper.

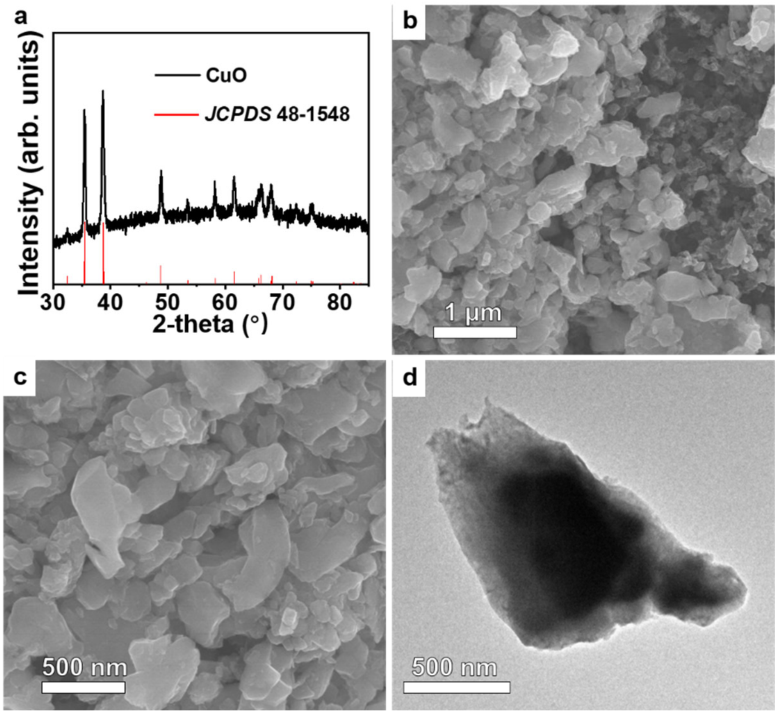

Supplementary Figure 30 | The structural characterizations of CuO. The XRD pattern (a), SEM images (b-c), and TEM image (d) of CuO. Source data are provided with this paper.

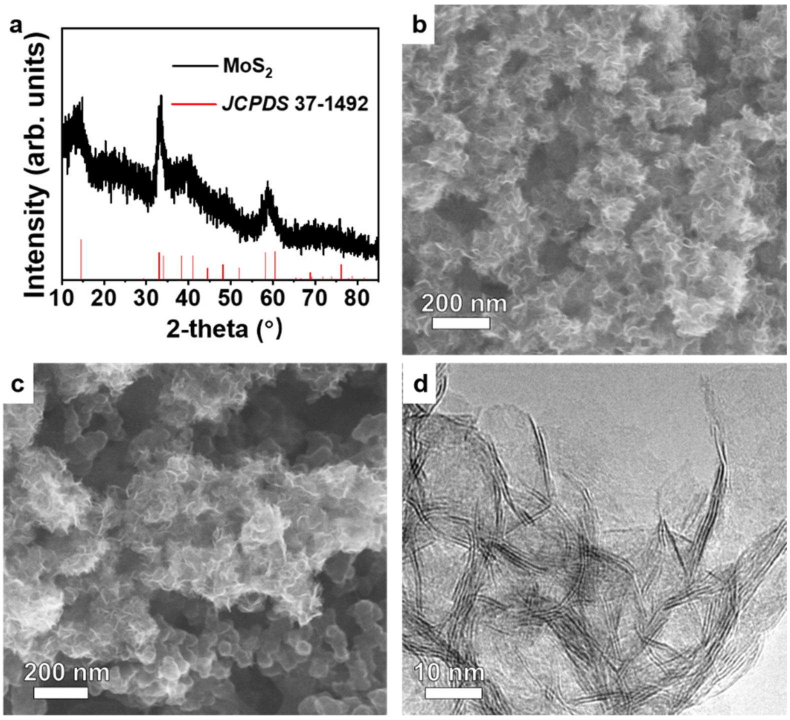

Supplementary Figure 31 | The structural characterizations of MoS_2. The XRD pattern (a), SEM images (b-c), and TEM image (d) of MoS_2. Source data are provided with this paper.

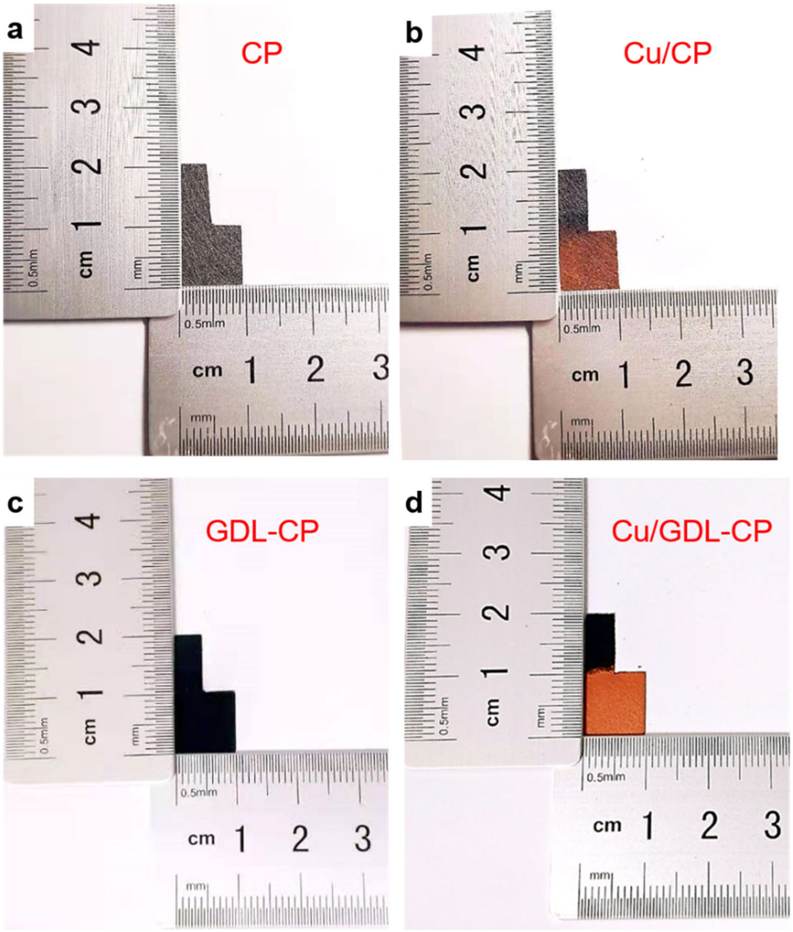

Supplementary Figure 32 | The photos of different working electrodes. Photos of carbon paper electrode (CP) (**a**), carbon paper loaded with Cu MPs (Cu/CP) (**b**), carbon paper coated with gas diffusion layer (GDL-CP) (**c**), and GDL-CP loaded with Cu MPs (Cu/GDL-CP) (**d**).

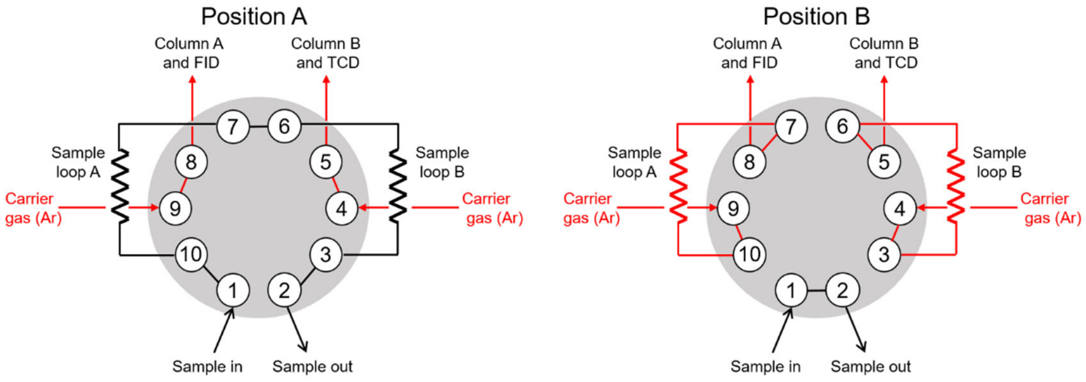

Supplementary Figure 33 | The Schematic diagram of a ten-port valve in two positions. Position A is standby mode. Position B is injection mode.

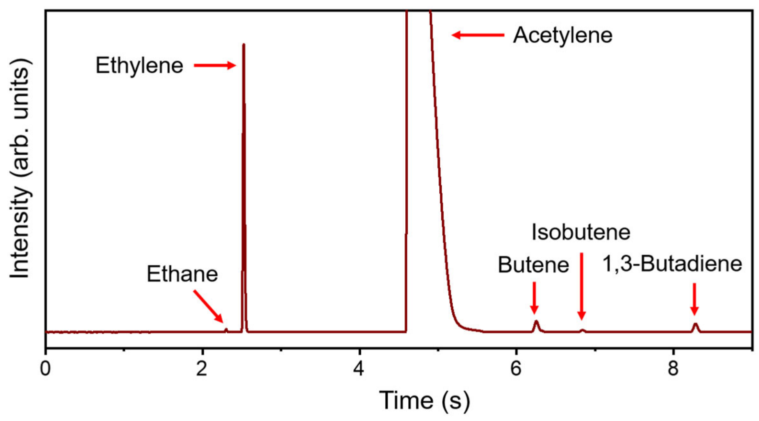

Supplementary Figure 34 | The analysis of gas products of E-HAE. A typical GC spectrum (measured with FID) of the E-HAE products in this work. Source data are provided with this paper.

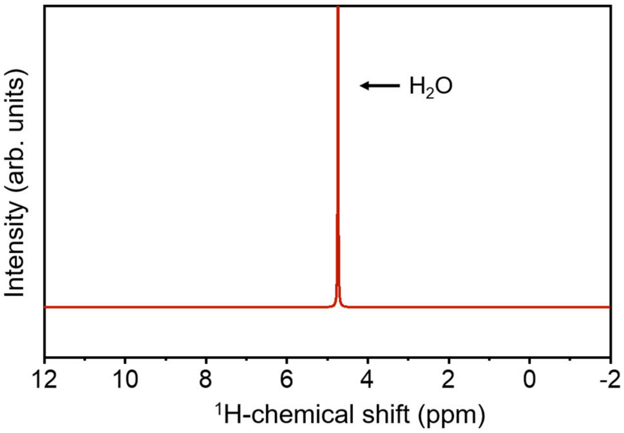

Supplementary Figure 35 | The analysis of liquid products of E-HAE. The NMR spectrum of the liquid phase after the E-HAE reaction. The experiment was conducted in 1 M KOH solution at -0.6 V vs. RHE, 25 ºC for 2 hours. Source data are provided with this paper.

Supplementary Table 1 | The E-HAE results of different catalysts tested in this work.

Catalysts	Potential (V vs. RHE)	geometric $j_{C_2H_4}$ (mA cm^{-2})	$FE_{C_2H_4}$
Cu MP	-0.5	17.1	77.6%
	-0.6	24.1	83.2%
	-0.7	26.7	68.1%
Cu Plate	-0.6	9.1	75.1%
	-0.7	16.7	36.4%
	-0.8	11.4	10.2%
CuO	-0.5	9.5	69.6%
	-0.6	15.2	48.3%
	-0.7	16.5	24.5%
Pt/C	-0.1	0.9	21.7%
	-0.2	3.6	19.5%
	-0.3	4.3	12.7%
Pd/C	-0.4	2.4	62.2%
	-0.5	7.1	43.8%
	-0.6	14.0	33.1%
Active Carbon	-0.5	0.2	11.4%
	-0.6	0.8	48.6%
	-0.7	0.8	41.4%
MoS_2	-0.3	0.6	24.2%
	-0.4	1.3	19.0%
	-0.5	0.76	8.9%
TiO_2	-0.6	0.3	4.4%
	-0.7	0.9	6.5%
	-0.8	0.9	2.7%

Supplementary Table 2 | Reaction free energies (ΔG) and activation energies (ΔG_{act}) in eV for the formation of C_2H_4 (g) on the C_2H_2-covered Cu(100), (110) and (111) surfaces at 0 V and -0.6 V vs RHE, 25 °C.

0 V vs RHE

Reaction	Cu(100)		Cu(110)		Cu(111)	
	ΔG	ΔG_{act}	ΔG	ΔG_{act}	ΔG	ΔG_{act}
C_2H_2 (g) → $*C_2H_2$	-0.17	-	-1.00	-	-1.39	-
$*C_2H_2 + H_2O + e^- \rightarrow *C_2H_3 + OH^-$	-0.36	0.79	-0.28	0.86	0.05	1.24
$*C_2H_3 + H_2O + e^- \rightarrow *C_2H_4 + OH^-$	-	-	-0.06	0.78	-0.25	0.52
$*C_2H_3 + H_2O + e^- \rightarrow C_2H_4$ (g) $+ OH^-$	-1.11	0.67				
$*C_2H_4 \rightarrow C_2H_4$ (g)			-0.30	-	-0.05	-

-0.6 V vs RHE

Reaction	Cu(100)		Cu(110)		Cu(111)	
	ΔG	ΔG_{act}	ΔG	ΔG_{act}	ΔG	ΔG_{act}
C_2H_2 (g) → $*C_2H_2$	-0.17	-	-1.00	-	-1.39	-
$*C_2H_2 + H_2O + e^- \rightarrow *C_2H_3 + OH^-$	-0.96	0.31	-0.88	0.43	-0.55	0.97
$*C_2H_3 + H_2O + e^- \rightarrow *C_2H_4 + OH^-$	-	-	-0.66	0.45	-0.85	0.10
$*C_2H_3 + H_2O + e^- \rightarrow C_2H_4$ (g) $+ OH^-$	-1.71	0.37				
$*C_2H_4 \rightarrow C_2H_4$ (g)			-0.30	-	-0.05	-

* refers to an adsorbed species.

Supplementary Table 3 | Reaction free energies (ΔG) and activation energies (ΔG_{act}) in eV for the formation of C_4H_6 (g) (First Pathway) on the C_2H_2-covered Cu(100), (110) and (111) surfaces at 0 V and -0.6 V vs. RHE, 25 °C.

0 V vs RHE

Reaction	Cu(100)		Cu(110)		Cu(111)	
	ΔG	ΔG_{act}	ΔG	ΔG_{act}	ΔG	ΔG_{act}
$2C_2H_2$ (g) → $2{*}C_2H_2$	-0.64	-	-2.03	-	-2.74	-
$*C_2H_2$ + ($*C_2H_2$ + H_2O + e^-) → $*C_2H_2$ + $*C_2H_3$ + OH^-	-0.36	0.79	-0.27	0.86	0.04	1.24
$*C_2H_2$ + $*C_2H_3$ → $*C_4H_5$	-1.72	0.36	-0.98	1.09	-0.59	1.17
$*C_4H_5$ + H_2O + e^- → $*C_4H_6$ + OH^-	-	-	-0.87	0.55	-0.52	0.26
$*C_4H_5$ + H_2O + e^- → C_4H_6 (g) + OH^-	-0.48	0.47				
$*C_4H_6$ → C_4H_6 (g)			0.95	-	0.61	-

-0.6 V vs RHE

Reaction	Cu(100)		Cu(110)		Cu(111)	
	ΔG	ΔG_{act}	ΔG	ΔG_{act}	ΔG	ΔG_{act}
$2C_2H_2$ (g) → $2{*}C_2H_2$	-0.64	-	-2.03	-	-2.74	-
$*C_2H_2$ + ($*C_2H_2$ + H_2O + e^-) → $*C_2H_2$ + $*C_2H_3$ + OH^-	-0.96	0.31	-0.87	0.43	-0.56	0.97
$*C_2H_2$ + $*C_2H_3$ → $*C_4H_5$	-1.72	0.36	-0.98	1.09	-0.59	1.17
$*C_4H_5$ + H_2O + e^- → $*C_4H_6$ + OH^-	-	-	-1.47	-	-1.12	-
$*C_4H_5$ + H_2O + e^- → C_4H_6 (g) + OH^-	-1.08	0.17				
$*C_4H_6$ → C_4H_6 (g)			0.95	-	0.61	-

* refers to an adsorbed species.

Supplementary Table 4 | Reaction free energies (ΔG) and activation energies (ΔG_{act}) in eV for the formation of C_4H_6 (g) (Second Pathway) on the C_2H_2-covered Cu(100), (110) and (111) surfaces at 0 V and -0.6 V vs. RHE, 25 °C.

0 V vs RHE

Reaction	Cu(100)		Cu(110)		Cu(111)	
	ΔG	ΔG_{act}	ΔG	ΔG_{act}	ΔG	ΔG_{act}
$2C_2H_2$ (g) → $2^*C_2H_2$	-0.64	-	-2.03	-	-2.74	-
$^*C_2H_2$ + ($^*C_2H_2$ + H_2O + e^-) → $^*C_2H_2$ + $^*C_2H_3$ + OH^-	-0.36	0.79	-0.27	0.86	0.04	1.24
($^*C_2H_2$ + H_2O + e^-) + $^*C_2H_3$ → $2^*C_2H_3$ + OH^-	-0.26	0.79	-0.30	0.86	0.01	1.24
$2^*C_2H_3$ → $^*C_4H_6$	-	-	-1.55	1.26	-1.12	0.84
$2^*C_2H_3$ → C_4H_6 (g)	-1.94	0.12				
$^*C_4H_6$ → C_4H_6 (g)			0.95	-	0.61	-

-0.6 vs RHE

Reaction	Cu(100)		Cu(110)		Cu(111)	
	ΔG	ΔG_{act}	ΔG	ΔG_{act}	ΔG	ΔG_{act}
$2C_2H_2$ (g) → $2^*C_2H_2$	-0.64	-	-2.03	-	-2.74	-
$^*C_2H_2$ + ($^*C_2H_2$ + H_2O + e^-) → $^*C_2H_2$ + $^*C_2H_3$ + OH^-	-0.96	0.31	-0.87	0.43	-0.56	0.97
($^*C_2H_2$ + H_2O + e^-) + $^*C_2H_3$ → $2^*C_2H_3$ + OH^-	-0.86	0.36	-0.90	0.41	-0.59	0.95
$2^*C_2H_3$ → $^*C_4H_6$	-	-	-1.55	1.26	-1.12	0.84
$2^*C_2H_3$ → C_4H_6 (g)	-1.94	0.12				
$^*C_4H_6$ → C_4H_6 (g)			0.95	-	0.61	-

* refers to an adsorbed species.

Supplementary Table 5 | Zero-point vibrational energies (E_{ZPE}), vibrational internal energy corrections (U_{vib}) and vibrational entropic corrections (TS_{vib}) at 25 °C for all the molecules and intermediates included in this study.

Free Molecules

Molecules	E_{ZPE}	$U_{vib+rot+trans}$	$TS_{vib+rot+trans}$
H_2 (g)	0.27	0.07	0.40
C_2H_2 (g)	0.71	0.08	0.62
C_2H_4 (g)	1.35	0.08	0.68
C_4H_6 (g)	2.26	0.13	0.85

Intermediates absorbed on clean Cu Surfaces

Intermediates	Cu(100)			Cu(110)			Cu(111)		
	E_{ZPE}	U_{vib}	TS_{vib}	E_{ZPE}	U_{vib}	TS_{vib}	E_{ZPE}	U_{vib}	TS_{vib}
*H	0.09	0.03	0.04	0.17	0.01	0.02	0.17	0.01	0.01
*C_2H_2	0.76	0.07	0.13	0.76	0.07	0.11	0.78	0.06	0.10
*C_2H_3	1.08	0.09	0.16	1.09	0.09	0.17	1.07	0.09	0.16
*C_2H_4	1.39	0.08	0.17	1.40	0.08	0.17	1.40	0.10	0.22
*C_4H_5	1.96	0.13	0.24	1.96	0.13	0.24	1.94	0.13	0.24
*C_4H_6	2.28	0.15	0.29	2.29	0.14	0.30	2.28	0.15	0.31

Intermediates absorbed on the C_2H_2-covered Cu Surfaces

Intermediates	Cu(100)			Cu(110)			Cu(111)		
	E_{ZPE}	U_{vib}	TS_{vib}	E_{ZPE}	U_{vib}	TS_{vib}	E_{ZPE}	U_{vib}	TS_{vib}
*H	0.15	0.01	0.01	0.18	0.01	0.01	0.18	0.01	0.02
*C_2H_2	0.80	0.06	0.09	0.76	0.07	0.11	0.78	0.06	0.09
*C_2H_3	1.11	0.08	0.14	1.09	0.08	0.14	1.07	0.09	0.18
*C_2H_4	-	-	-	1.33	0.10	0.16	1.38	0.10	0.21
*C_4H_5	1.99	0.14	0.28	1.98	0.14	0.26	1.95	0.12	0.21
*C_4H_6	-	-	-	2.28	0.14	0.26	2.28	0.15	0.28

The footnotes 'rot' and 'trans' mean rotational and translational, respectively.

Supplementary Table 6 | Zero-point vibrational energies (E_{ZPE}) and vibrational internal energy corrections (U_{vib}) at 25 °C for the initial and transition states (TS).

Cu (100)

Reaction	E_{ZPE}		U_{vib}	
	IS	TS	IS	TS
$*C_2H_2 + H_2O + e^- \rightarrow *C_2H_3 + OH^-$	1.45	1.27	0.11	0.11
$*C_2H_3 + H_2O + e^- \rightarrow C_2H_4\,(g) + OH^-$	1.78	1.67	0.12	0.11
$*C_2H_2 + *C_2H_3 \rightarrow *C_4H_5$	1.91	1.89	0.13	0.12
$2*C_2H_3 \rightarrow C_4H_6\,(g)$	2.21	2.20	0.16	0.15
$*C_4H_5 + H_2O + e^- \rightarrow C_4H_6\,(g) + OH^-$	2.65	2.53	0.19	0.17

Cu (110)

Reaction	E_{ZPE}		U_{vib}	
	IS	TS	IS	TS
$*C_2H_2 + H_2O + e^- \rightarrow *C_2H_3 + OH^-$	1.43	1.32	0.12	0.09
$*C_2H_3 + H_2O + e^- \rightarrow *C_2H_4 + OH^-$	1.77	1.63	0.13	0.11
$*C_2H_2 + *C_2H_3 \rightarrow *C_4H_5$	1.85	1.85	0.15	0.13
$2*C_2H_3 \rightarrow *C_4H_6$	2.18	2.17	0.16	0.15
$*C_4H_5 + H_2O + e^- \rightarrow *C_4H_6 + OH^-$	2.66	2.54	0.18	0.16

Cu (111)

Reaction	E_{ZPE}		U_{vib}	
	IS	TS	IS	TS
$*C_2H_2 + H_2O + e^- \rightarrow *C_2H_3 + OH^-$	1.46	1.37	0.11	0.09
$*C_2H_3 + H_2O + e^- \rightarrow *C_2H_4 + OH^-$	1.75	1.63	0.14	0.11
$*C_2H_2 + *C_2H_3 \rightarrow *C_4H_5$	1.85	1.85	0.15	0.14
$2*C_2H_3 \rightarrow *C_4H_6$	2.14	2.15	0.19	0.16
$*C_4H_5 + H_2O + e^- \rightarrow *C_4H_6 + OH^-$	2.60	2.52	0.17	0.15

Supplementary Table 7 | Zero-point vibrational energy changes (ΔE_{ZPE}) and vibrational internal energy correction changes (ΔU_{vib}) at 25 °C between the transition states and the initial states.

Cu (100)

Reaction	ΔE_{ZPE}	ΔU_{vib}
$*C_2H_2 + H_2O + e^- \rightarrow *C_2H_3 + OH^-$	-0.18	0.00
$*C_2H_3 + H_2O + e^- \rightarrow C_2H_4\ (g) + OH^-$	-0.11	-0.01
$*C_2H_2 + *C_2H_3 \rightarrow *C_4H_5$	-0.02	-0.01
$2*C_2H_3 \rightarrow C_4H_6\ (g)$	-0.01	-0.01
$*C_4H_5 + H_2O + e^- \rightarrow C_4H_6\ (g) + OH^-$	-0.12	-0.02

Cu (110)

Reaction	ΔE_{ZPE}	ΔU_{vib}
$*C_2H_2 + H_2O + e^- \rightarrow *C_2H_3 + OH^-$	-0.11	-0.03
$*C_2H_3 + H_2O + e^- \rightarrow *C_2H_4 + OH^-$	-0.14	-0.02
$*C_2H_2 + *C_2H_3 \rightarrow *C_4H_5$	0.00	-0.02
$2*C_2H_3 \rightarrow *C_4H_6$	-0.01	-0.01
$*C_4H_5 + H_2O + e^- \rightarrow *C_4H_6 + OH^-$	-0.12	-0.02

Cu (111)

Reaction	ΔE_{ZPE}	ΔU_{vib}
$*C_2H_2 + H_2O + e^- \rightarrow *C_2H_3 + OH^-$	-0.09	-0.02
$*C_2H_3 + H_2O + e^- \rightarrow *C_2H_4 + OH^-$	-0.12	-0.03
$*C_2H_2 + *C_2H_3 \rightarrow *C_4H_5$	0.00	-0.01
$2*C_2H_3 \rightarrow *C_4H_6$	0.01	-0.03
$*C_4H_5 + H_2O + e^- \rightarrow *C_4H_6 + OH^-$	-0.08	-0.02

Supplementary Table 8 | Calculated vibrational frequencies in cm^{-1} for the free molecules used in this study.

Free Molecule	Vibrational frequencies (cm^{-1})
H_2 (g)	4306.5, 131.6, 69.3
C_2H_2 (g)	3448.1, 3354.8, 2012.9, 729.2, 727.8, 621.9, 615.5, 115.6, 91.3
C_2H_4 (g)	3168.3, 3135.1, 3089.8, 3066.2, 1640.2, 1429.8, 1359.1, 1205.0, 1041.6, 943.7, 937.8, 817.3, 123.0, 86.4, 14.4
C_4H_6 (g)	3171.8, 3168.8, 3085.9, 3084.0, 3073.3, 3059.7, 1653.4, 1604.8, 1432.4, 1381.9, 1284.8, 1280.8, 1204.5, 1026.2, 981.2, 970.0, 909.0, 895.4, 889.4, 764.8, 536.5, 507.7, 282.6, 204.3, 130.4, 122.2

Supplementary Table 9 | Calculated vibrational frequencies in cm^{-1} for the intermediates on clean Cu(100) surface.

Intermediates	Vibrational frequencies (cm^{-1})
*H	1236.5, 1070.2, 262.5
*C_2H_2	2994.3, 2965.5, 1312.9 , 1153.0, 1002.7, 927.2, 535.2, 465.6, 377.8, 265.0, 184.0, 107.2
*C_2H_3	3066.6, 2962.0, 2954.4, 1478.7, 1327.0, 1213.9, 993.3, 948.8, 912.2, 491.0, 417.2, 264.0, 201.6, 156.5, 66.2
*C_2H_4	3157.6, 3133.6, 3060.4, 3048.1, 1515.6, 1399.1, 1265.9, 1179.0, 963.1, 899.6, 894.7, 792.3, 519.4, 292.4, 196.1, 95.5, 49.0
*C_4H_5	3152.8, 3063.8, 3016.9, 2982.9, 2933.2, 1486.5, 1415.3, 1327.4, 1251.5, 1210.8, 1184.9, 1056.3, 915.1, 905.8, 884.9, 841.4, 769.1, 665.3, 551.3, 439.5, 377.0, 320.3, 291.8, 224.9, 182.2, 97.5, 65.0
*C_4H_6	3159.4, 3157.1, 3075.7, 3071.9, 3047.7, 3034.8, 1494.8, 1455.2, 1406.1, 1303.8, 1218.5, 1210.2, 1199.1, 984.2, 937.6, 878.2, 861.6, 837.5, 790.4, 719.8, 672.3, 492.1, 399.7, 323.2, 271.2, 251.3, 218.7, 81.3, 75.9, 64.2

Supplementary Table 10 | Calculated vibrational frequencies in cm^{-1} for the intermediates on clean Cu(110) surface.

Intermediates	Vibrational frequencies (cm^{-1})
*H	1287.3, 1182.2, 281.4
$*C_2H_2$	2964.2, 2935.1, 1264.4, 1147.7, 1037.2, 867.6, 505.6, 455.9, 316.4, 250.7, 224.8, 215.5
$*C_2H_3$	3071.3, 3011.1, 2978.2, 1481.7, 1332.8, 1192.9, 988.1, 946.0, 916.6, 494.5, 429.7, 271.8, 197.9, 136.8, 64.9
$*C_2H_4$	3159.5, 3137.3, 3056.4, 3048.9, 1484.2, 1406.9, 1217.2, 1200.3, 913.4, 891.5, 852.8, 832.3, 581.1, 362.8, 248.3, 76.3, 43.0
$*C_4H_5$	3145.6, 3054.1, 3021.6, 2996.3, 2939.0, 1488.5, 1418.4, 1331.4, 1247.9, 1216.2, 1192.4, 1054.5, 910.3, 904.9, 875.4, 838.3, 766.1, 654.9, 552.9, 457.4, 374.8, 338.2, 313.7, 236.5, 191.9, 92.8, 61.6
$*C_4H_6$	3154.7, 3154.3, 3066.2, 3064.3, 3057.2, 3043.0, 1490.4, 1447.5, 1409.1, 1306.7, 1218.9, 1211.9, 1194.7, 995.5, 960.5, 881.5, 879.4, 847.9, 811.6, 730.8, 695.8, 495.3, 422.1, 356.3, 293.7, 272.3, 269.7, 88.6, 71.8, 27.2

Supplementary Table 11 | Calculated vibrational frequencies in cm^{-1} for the intermediates on the clean Cu(111) surface.

Intermediates	Vibrational frequencies (cm^{-1})
*H	1087.0, 827.9, 824.1
*C_2H_2	3038.9, 3018.7, 1261.0, 1120.9, 952.2, 897.8, 672.6, 474.2, 381.1, 274.5, 249.0, 245.3
*C_2H_3	3102.3, 3018.0, 2795.3, 1462.3, 1291.8, 1225.1, 959.8, 913.4, 872.5, 496.5, 375.4, 267.9, 197.6, 146.1, 88.3
*C_2H_4	3162.5, 3135.4, 3068.2, 3051.0, 1485.2, 1398.1, 1239.9, 1188.6, 921.0, 900.3, 872.7, 817.6, 567.0, 333.0, 197.2, 100.2, 74.6, 53.5
*C_4H_5	3155.8, 3058.7, 3021.8, 2997.4, 2911.3, 1461.9, 1396.2, 1303.3, 1215.3, 1206.6, 1176.7, 1057.9, 894.5, 876.2, 858.6, 786.2, 716.5, 661.3, 536.4, 418.4, 392.3, 319.0, 283.9, 217.2, 195.3, 119.8, 63.0
*C_4H_6	3160.3, 3152.2, 3066.2, 3058.9, 3034.4, 3019.7, 1480.4, 1439.3, 1404.8, 1291.9, 1203.0, 1193.5, 1187.1, 991.7, 919.0, 886.1, 879.8, 835.1, 829.9, 729.1, 693.0, 488.2, 423.2, 361.7, 275.0, 270.8, 243.7, 88.9, 69.8, 18.9

Supplementary Table 12 | Calculated vibrational frequencies in cm^{-1} for the intermediates on the C_2H_2-covered Cu(100) surface.

Intermediates	Vibrational frequencies (cm^{-1})
*H	1115.3, 696.1, 657.6
*C_2H_2	3018.0, 2990.0, 1304.1, 1106.8, 958.5, 907.6, 731.3, 506.8, 385.5, 357.7, 334.7, 220.7
*C_2H_3	3110.6, 3039.0, 3014.4, 1519.8, 1359.4, 1205.8, 1021.6, 973.2, 898.0, 460.3, 359.5, 287.1, 230.6, 212.7, 127.1
*C_2H_4	-
*C_4H_5	3157.4, 3072.0, 3033.1, 3007.5, 2978.5, 1608.0, 1487.3, 1388.4, 1280.9, 1268.9, 1243.9, 1094.6, 1012.3, 925.5, 914.7, 878.0, 765.2, 590.1, 563.4, 423.6, 299.6, 253.0, 216.8, 170.6, 128.2, 107.6, 29.2
*C_4H_6	-

Supplementary Table 13 | Calculated vibrational frequencies in cm^{-1} for the intermediates on the C_2H_2-covered Cu(110) surface.

Intermediates	Vibrational frequencies (cm^{-1})
*H	1343.5, 780.9, 725.9
*C_2H_2	2941.2, 2909.0, 1275.6, 1147.0, 1052.4, 893.0, 548.6, 447.6, 303.2, 234.6, 229.4, 213.5
*C_2H_3	3102.8, 3020.2, 2896.3, 1455.9, 1269.8, 1213.4, 988.6, 949.6, 917.3, 578.2, 340.9, 286.7, 207.5, 184.7, 137.2
*C_2H_4	2991.3, 2966.6, 2935.8, 2927.7, 1357.9, 1322.6, 1190.6, 1088.4, 999.7, 948.4, 769.1, 508.1, 386.8, 331.6, 225.7, 179.2, 169.7, 162.3
*C_4H_5	3161.8, 3074.1, 3055.7, 3024.0, 2957.2, 1588.8, 1449.0, 1374.9, 1277.7, 1252.4, 1210.5, 1063.5, 995.7, 940.9, 910.7, 876.5, 800.3, 610.1, 574.1, 447.4, 364.8, 263.8, 225.9, 193.9, 116.7, 97.4, 76.7
*C_4H_6	3162.5, 3142.1, 3083.3, 3069.6, 3010.7, 3001.5, 1495.3, 1447.3, 1395.7, 1306.8, 1231.4, 1208.9, 1204.4, 998.3, 939.1, 881.3, 866.4, 845.9, 815.4, 726.3, 668.0, 506.3, 390.1, 339.1, 304.3, 275.7, 213.4, 119.2, 99.2, 80.4

Supplementary Table 14 | Calculated vibrational frequencies in cm^{-1} for the intermediates on the C_2H_2-covered Cu(111) surface.

Intermediates	Vibrational frequencies (cm^{-1})
*H	1468.6, 1031.1, 342.2
*C_2H_2	3024.6, 3001.8, 1290.3, 1096.7, 926.1, 880.0, 665.4, 478.1, 386.6, 283.3, 277.6, 258.2
*C_2H_3	3093.1, 3011.4, 2847.0, 1497.6, 1352.7, 1256.6, 987.2, 954.8, 901.2, 358.4, 350.8, 216.6, 199.6, 113.7, 87.7
*C_2H_4	3204.4, 3111.0, 3056.1, 2971.7, 1461.8, 1375.4, 1192.4, 1178.1, 876.0, 864.5, 810.9, 791.5, 550.6, 334.5, 192.2, 171.4, 71.1, 58.9
*C_4H_5	3156.7, 3067.7, 3025.4, 2911.2, 2822.6, 1450.0, 1399.1, 1285.1, 1227.5, 1198.1, 1159.9, 1064.3, 928.0, 900.8, 882.3, 819.8, 742.6, 676.4, 558.4, 439.5, 377.9, 317.3, 308.5, 246.6, 199.7, 137.5, 117.6
*C_4H_6	3182.3, 3176.7, 3074.8, 3073.8, 3051.1, 3046.8, 1490.4, 1445.2, 1401.0, 1298.2, 1223.4, 1194.7, 1189.0, 987.8, 905.4, 880.7, 863.1, 824.1, 811.9, 729.0, 682.1, 490.0, 423.6, 353.8, 285.9, 230.0, 204.2, 135.0, 75.7, 51.4

Supplementary Table 15 | Calculated vibrational frequencies in cm^{-1} for the reacting species at the transition states on the C_2H_2-covered Cu(100) surface. The frequencies in the brackets denote the imaginary frequencies of the transition states.

Reaction	Vibrational frequencies (cm^{-1})
$^*C_2H_2 + H_2O + e^- \rightarrow {}^*C_2H_3 + OH^-$	3145.0, 2909.8, 2384.7, 1490.6, 1434.8, 1259.3, 1169.8, 1068.0, 955.1, 906.9, 861.1, 519.2, 506.5, 390.5, 368.0, 270.9, 221.9, 220.9, 191.4, 158.0, [1073.9]
$^*C_2H_3 + H_2O + e^- \rightarrow C_2H_4$ (g) + OH^-	3057.4, 3011.3, 2996.1, 2741.9, 1604.6, 1498.9, 1384.0, 1280.8, 1192.4, 1167.4, 1029.0, 974.2, 922.5, 853.0, 723.2, 659.6, 481.6, 367.0, 257.5, 231.8, 205.3, 145.7, 113.5, [85.0]
$^*C_2H_2 + {}^*C_2H_3 \rightarrow {}^*C_4H_5$	3151.6, 3132.4, 3062.5, 3040.8, 2975.8, 1508.8, 1344.8, 1228.4, 1198.2, 1163.9, 1074.0, 1005.0, 957.6, 865.1, 838.7, 727.8, 510.0, 486.9, 409.1, 392.4, 361.5, 310.0, 272.2, 211.1, 171.0, 137.3, [398.1]
$2^*C_2H_3 \rightarrow C_4H_6$ (g)	3146.5, 3145.4, 3108.0, 3099.2, 3041.7, 3040.7, 1509.8, 1498.3, 1346.8, 1343.8, 1207.0, 1187.1, 1028.6, 981.0, 950.2, 945.5, 834.7, 829.7, 499.7, 495.2, 378.6, 363.5, 311.3, 301.0, 236.3, 200.9, 157.6, 152.5, 140.9, [368.3]
$^*C_4H_5 + H_2O + e^- \rightarrow C_4H_6$ (g) + OH^-	3166.3, 3086.4, 3069.9, 3055.7, 2978.6, 2970.8, 1591.8, 1453.3, 1390.2, 1371.1, 1291.3, 1254.8, 1224.3, 1200.9, 1102.6, 1041.4, 1001.9, 968.3, 936.6, 926.5, 901.5, 821.5, 634.2, 583.3, 424.3, 393.0, 329.8, 312.4, 262.2, 248.1, 209.9, 205.3, 130.8, 126.2, 63.4, [1315.8]

Supplementary Table 16 | Calculated vibrational frequencies in cm^{-1} for the reacting species at the transition states on the C_2H_2-covered Cu(110) surface. The frequencies in the brackets denote the imaginary frequencies of the transition states.

Reaction	Vibrational frequencies (cm^{-1})
$^*C_2H_2 + H_2O + e^- \rightarrow {^*C_2H_3} + OH^-$	2881.3, 2711.6, 2628.5, 1425.8, 1275.5, 1248.8, 1232.6, 1188.8, 1066.8, 1017.8, 872.3, 624.4, 606.9, 518.6, 422.1, 341.6, 335.4, 297.1, 268.0, 241.7, [1175.4]
$^*C_2H_3 + H_2O + e^- \rightarrow {^*C_2H_4} + OH^-$	3247.7, 3081.9, 2974.1, 2839.8, 1516.3, 1431.7, 1265.2, 1220.3, 1156.2, 1149.4, 990.3, 908.2, 857.0, 847.1, 563.9, 551.7, 397.3, 318.4, 275.4, 254.5, 208.2, 185.5, 117.2, [756.0]
$^*C_2H_2 + {^*C_2H_3} \rightarrow {^*C_4H_5}$	3115.7, 3111.8, 3079.7, 3025.1, 2956.2, 1511.4, 1441.2, 1251.1, 1204.4, 1013.7, 974.2, 925.4, 901.6, 815.0, 759.8, 659.4, 602.7, 493.9, 384.5, 366.4, 349.4, 261.3, 212.9, 194.6, 155.3, 122.5, [150.6]
$2^*C_2H_3 \rightarrow {^*C_4H_6}$	3147.8, 3118.8, 3048.4, 3027.7, 2930.1, 2677.0, 1505.1, 1443.7, 1355.5, 1275.4, 1240.4, 1221.6, 1076.1, 999.9, 974.5, 919.6, 891.3, 870.0, 662.6, 464.2, 368.3, 341.2, 309.1, 288.1, 222.3, 207.6, 162.0, 139.4, 120.8, [249.8]
$^*C_4H_5 + H_2O + e^- \rightarrow {^*C_4H_6} + OH^-$	3154.7, 3079.3, 3050.6, 2939.7, 2923.3, 2742.8, 1610.5, 1501.9, 1434.4, 1341.5, 1264.0, 1230.9, 1227.1, 1195.9, 1107.0, 1082.6, 1035.4, 986.4, 938.3, 894.6, 857.3, 798.3, 713.9, 626.7, 561.5, 425.0, 408.1, 358.8, 323.1, 301.0, 290.8, 205.8, 144.2, 133.5, 93.2, [343.4]

Supplementary Table 17 | Calculated vibrational frequencies in cm^{-1} for the reacting species at the transition states on the C_2H_2-covered Cu(111) surface. The frequencies in the brackets denote the imaginary frequencies of the transition states.

Reaction	Vibrational frequencies (cm^{-1})
$*C_2H_2 + H_2O + e^- \rightarrow *C_2H_3 + OH^-$	3282.5, 3004.8, 2983.8, 1905.5, 1288.1, 1169.8, 1134.2, 1112.3, 969.1, 914.4, 882.4, 664.0, 509.1, 476.8, 381.5, 353.1, 292.7, 263.8, 236.7, 204.0, [298.9]
$*C_2H_3 + H_2O + e^- \rightarrow *C_2H_4 + OH^-$	3061.5, 2983.5, 2899.2, 2819.2, 1570.5, 1406.2, 1272.7, 1170.6, 1143.4, 1100.1, 1000.7, 946.9, 920.5, 831.1, 695.8, 542.6, 392.7, 352.6, 306.5, 282.9, 231.7, 195.7, 155.3, [1000.0]
$*C_2H_2 + *C_2H_3 \rightarrow *C_4H_5$	3101.7, 3080.5, 3029.0, 3027.1, 2992.2, 1483.2, 1341.0, 1250.1, 1198.9, 1090.0, 983.2, 956.2, 943.7, 871.3, 866.1, 706.8, 471.6, 435.1, 385.2, 366.4, 332.6, 285.8, 228.0, 202.4, 157.9, 41.2, [154.6]
$2*C_2H_3 \rightarrow *C_4H_6$	3087.7, 3077.5, 3046.2, 3006.2, 2998.1, 2975.8, 1485.0, 1467.2, 1334.6, 1321.0, 1222.6, 1208.8, 1002.4, 963.8, 955.9, 946.9, 859.6, 858.0, 486.2, 459.6, 409.8, 344.6, 253.6, 248.7, 207.7, 183.7, 138.6, 118.6, 86.0, [53.7]
$*C_4H_5 + H_2O + e^- \rightarrow *C_4H_6 + OH^-$	3216.8, 3174.8, 3082.1, 3066.8, 2909.9, 2621.7, 1559.9, 1460.0, 1392.7, 1286.7, 1260.8, 1205.3, 1199.0, 1169.6, 1117.5, 1061.2, 972.7, 910.8, 888.9, 868.9, 856.0, 822.0, 684.3, 613.4, 518.6, 460.8, 400.5, 361.2, 338.0, 275.7, 245.3, 224.3, 196.4, 141.9, 130.5, [907.8]

Supplementary Table 18 | Calculated value for U^0.

Reaction	Cu(100)	Cu(110)	Cu(111)
$*C_2H_2 \rightarrow *C_2H_3$	0.36	0.27	-0.05
$*C_2H_3 \rightarrow *C_2H_4$	-	0.07	0.25
$*2C_2H_2 \rightarrow *C_2H_2 + *C_2H_3$	0.36	0.27	-0.05
$*C_2H_2 + *C_2H_3 \rightarrow 2*C_2H_3$	0.26	0.30	-0.01
$*C_4H_5 \rightarrow *C_4H_6$	-	0.87	0.52

Supplementary References

1. Hahn, T & Looijenga-Vos, A. *International table for crystallography*. **A**, 17-41 (2016).
2. Brisard, G. M., Zenati, E., Gasteiger, H. A., Markovic, N. & Ross, P. N. Underpotential Deposition of Lead on Copper(111): A Study Using a Single-Crystal Rotating Ring Disk Electrode and ex Situ Low-Energy Electron Diffraction and Scanning tunneling Microscopy. Langmuir 11, 2221-2230 (1995).
3. Brisard, G. M., Zenati, E., Gasteiger, H. A., Markovic, N. M. & Ross, P. N. Underpotential deposition of lead on Cu(100) in the presence of chloride: Ex-situ low-energy electron diffraction, auger electron spectroscopy, and electrochemical studies. Langmuir 13, 2390-2397 (1997).
4. Moffat, T. P. Oxidative Chloride Adsorption and Lead Upd on Cu(100): Investigations into Surfactant-Assisted Epitaxial Growth. J. Phys. Chem. B 102, 10020-10026 (1998).
